# *In vitro* activity assessment of cefiderocol against Enterobacterales, *Pseudomonas aeruginosa,* and *Acinetobacter* spp., including β-lactam nonsusceptible molecularly characterized isolates, collected from 2020 to 2021 in the United States and European hospitals

**DOI:** 10.1128/spectrum.01474-24

**Published:** 2024-10-10

**Authors:** John H. Kimbrough, Joshua M. Maher, Helio S. Sader, Mariana Castanheira, Rodrigo E. Mendes

**Affiliations:** 1 JMI Laboratories/Element Iowa City, North Liberty, Iowa, USA; Universidad de Buenos Aires, Buenos Aires, Argentina

**Keywords:** *Acinetobacter baumannii*, cefiderocol, CRE, multidrug resistance, *Pseudomonas aeruginosa*

## Abstract

This study reports the activity of cefiderocol against Enterobacterales, *Pseudomonas aeruginosa*, and *Acinetobacter* spp. isolates collected from the United States and Europe, including Israel and Turkey, from 2020 to 2021. Among Enterobacterales, 2.8% were carbapenem nonsusceptible (CNSE); cefiderocol inhibited 96.6%/85.1% [Clinical Laboratory Standards Institute (CLSI)/European Committee on Antimicrobial Susceptibility Testing (EUCAST) breakpoints] of these isolates. Imipenem-relebactam, meropenem-vaborbactam, and ceftazidime-avibactam displayed susceptibilities lower than cefiderocol against CNSE isolates (67.4–84.6% susceptible, CLSI). Cefiderocol was the only agent active against CNSE isolates carrying metallo-β-lactamase (MBL) carbapenemase or multiple carbapenemase genes (84.6%–92.3% susceptible, CLSI). Approximately 18% of carbapenem-susceptible *Escherichia coli*, *Klebsiella pneumoniae*, and *Proteus mirabilis* carried extended-spectrum-β-lactamases and/or plasmid-borne AmpC-encoding genes; cefiderocol inhibited 99.8%–100.0% (CLSI) of these genotypic groups. Multi-drug resistance (MDR) phenotypes were observed in 16.9% and 52.5% of *P. aeruginosa* and *A. baumannii-calcoaceticus* isolates, respectively. Carbapenemase genes were rare (4.9%) among cephalosporin and/or carbapenem nonsusceptible *P. aeruginosa*, compared to 87.6% carriage in *A. baumannii-calcoaceticus*, respectively. Against the MDR and carbapenemase-carrying *P. aeruginosa* and *A. baumannii-calcoaceticus* subsets, cefiderocol was active against 98.6%/98.7% and 97.1%/97.4% (CLSI), respectively. Only 69 isolates (0.3%) across all species groups were identified as cefiderocol nonsusceptible per CLSI criteria (>4 mg/L). Cefiderocol was the most active agent *in vitro* against Enterobacterales, *P. aeruginosa,* and *Acinetobacter* spp., with uniform activity against all phenotypic- and genotypic-resistant subsets. Coupled with the low incidence of nonsusceptibility observed across species groups, these results demonstrate cefiderocol as an important option for treating infections caused by pathogens with diverse mechanisms of resistance in US and European hospitals.

**IMPORTANCE:**

The worldwide spread of multi-drug-resistant *Pseudomonas aeruginosa* and carbapenem-resistant Enterobacterales and *Acinetobacter* spp. poses a serious challenge in healthcare settings as infections caused by these organisms are commonly refractory to many frontline therapeutic agents. Multiple global health organizations highlighted these pathogens as critical priorities for new antibiotic development, thus necessitating continued surveillance of the activities of currently available antimicrobial agents and circulating mechanisms of resistance. To meet this need, this study phenotypically and genotypically characterized priority Gram-negative pathogens collected from patients in US and European hospitals to examine the activity of cefiderocol and other currently available treatment options, including carbapenems and β-lactam-β-lactamase inhibitor combinations. The results presented here provide a detailed perspective to healthcare practitioners of cefiderocol’s broad applicability, manifested in high activity and low nonsusceptibility rates, across phenotypic and genotypic organism groups relative to other agents and further support its use against the most intransigent infections.

## INTRODUCTION

The continual increase in antibiotic resistance poses a global threat to human health. A report from the Centers for Disease Control and Prevention (CDC) noted more than 2.8 million antibiotic-resistant infections occur in United States (US) medical centers each year, and more than 35,000 patients die as a result (1). This report identified carbapenem-resistant Enterobacterales (CRE) and carbapenem-resistant *Acinetobacter* spp. as urgent threats. Multi-drug-resistant (MDR) *Pseudomonas aeruginosa* was identified by the CDC report as a serious threat, and *P. aeruginosa* isolates with a “difficult-to-treat” resistance (DTR) phenotype (2), nonsusceptible to all frontline therapeutic agents, recently received updated guidance for treatment from the Infectious Disease Society of America (3). The World Health Organization has also classified these three pathogen groups as critical priorities for the development of new antibiotics (4). Infections caused by these pathogens are difficult to treat due to limited therapeutic options, and often the administration of appropriate therapy is delayed, further contributing to increased patient morbidity, mortality, and additional negative outcomes (5).

Cefiderocol is comprised of a synthetic cephalosporin moiety and an iron-binding, catechol-type siderophore which facilitates drug entry into the bacterial cell using intrinsic siderophore transporters. After reaching the periplasmic space, the cephalosporin component binds to penicillin-binding protein 3 and inhibits bacterial cell wall synthesis (6). In addition to this unique cell entry mechanism, cefiderocol possesses improved hydrolytic stability against extended-spectrum β-lactamases (ESBLs) and carbapenemases (7–9) and demonstrates activity in isolates with altered cell permeability (e.g., porin mutants and efflux pumps). Cefiderocol was approved by the US Food and Drug Administration (FDA) in November 2019 for the treatment of complicated urinary tract infections (UTIs), including pyelonephritis, caused by Gram-negative organisms and further approved in September 2020 for the treatment of hospital-acquired bacterial pneumonia and ventilator-associated bacterial pneumonia caused by Enterobacterales, *P. aeruginosa*, and *A. baumannii-calcoaceticus* species complex strains (10). Likewise, cefiderocol was approved by the European Medicines Agency in April 2020 for the treatment of infections caused by aerobic, Gram-negative organisms when therapeutic options are limited (10).

The *in vitro* activity of cefiderocol was monitored against Gram-negative pathogens causing infections in patients hospitalized in the US and European medical centers during 2020 and 2021 as part of the SENTRY Antimicrobial Surveillance Program. This study reports on the activity of cefiderocol and various comparator agents against these organisms collected from the US, European countries, and adjacent regions, including β-lactam-resistant clinical isolates molecularly characterized for β-lactamase gene content, following cefiderocol’s market introduction.

## RESULTS

### Enterobacterales

A total of 16,065 clinical Enterobacterales isolates were included as part of the global SENTRY surveillance program monitoring the activity of cefiderocol during 2020–2021. Imipenem and/or meropenem nonsusceptibility was observed in 2.8% (442/16,065) of all Enterobacterales (Table 1), with proportions of 4.1% (317/7,739) and 1.5% (125/8,326) observed within isolates from Europe and the USA, respectively (data not shown). Breakdowns of infection type, geographic origin, and species/species group across study years for carbapenem nonsusceptible Enterobaterales are displayed in Tables S1 and S2. Most carbapenem-nonsusceptible isolates carried *bla*_KPC_ (*n* = 187; 42.4%) as the sole carbapenemase gene, followed by OXA-48-like (*n* = 79; 17.9%) and metallo-β-lactamase genes (*n* = 52; 11.8%); 13 (2.9%) carbapenem nonsusceptible isolates, all *K. pneumoniae,* had dual carbapenemases (8 *bla*_KPC_ and MBL-encoding gene; 5 *bla*_OXA-48-like_ and MBL-encoding gene). Cefiderocol inhibited 96.6% of Enterobacterales isolates nonsusceptible to carbapenems at ≤4 mg/L (CLSI|FDA breakpoint for susceptibility) (Table 1), and cefiderocol (MIC_50/90_, 1/4 mg/L) and ceftazidime-avibactam (MIC_50/90_, 1/>32 mg/L; 84.6% susceptible) were the most active agents against these isolates (Table 2). Imipenem-relebactam (MIC_50/90_, 0.5/>8 mg/L) and meropenem-vaborbactam (MIC_50/90_, 0.5/>8 mg/L) showed susceptibility of 67.4%–74.7% and 73.8%–78.1% per CLSI|FDA and EUCAST criteria, respectively (Table 2) (11–13).

**TABLE 1:** MIC distribution of cefiderocol obtained against Enterobacterales and prominent genotypic subsets collected in 2020–2021 from US and European hospitals

Organism/group (no. of isolates)	No. and cumulative % of isolates inhibited at MIC (mg/L) of:														MIC_50_	MIC_90_
	≤0.004	0.008	0.015	0.03	0.06	0.12	0.25	0.5	1	2	4	8	16	>16		
All Enterobacterales																
Carbapenem-S^*a*^ (15,623)	395 2.5	1,190 10.1	1,956 22.7	2,076 36.0	2,566 52.4	2,849 70.6	2,302 85.3	1,168 92.8	671 97.1	319 99.2	116 99.9	11 > 99.9	2 > 99.9	2 100.0	0.06	0.5
Carbapenem-NS^*a*^^,^^*b*^ (442)		4 0.9	2 1.4	19 5.7	23 10.9	44 20.8	55 33.3	72 49.5	73 66.1	84 85.1	51 96.6	12 99.3	0 99.3	3 100.0	1	4
KPC^*c*^ (187)		2 1.1	0 1.1	9 5.9	4 8.0	20 18.7	34 36.9	30 52.9	30 69.0	41 90.9	15 98.9	1 99.5	0 99.5	1 100.0	0.5	2
OXA-48-like^*d*^ (79)		1 1.3	1 2.5	5 8.9	7 17.7	10 30.4	13 46.8	14 64.6	13 81.0	9 92.4	6 100.0				0.5	2
MBL^*e*^ (52)					1 1.9	1 3.8	1 5.8	2 9.6	11 30.8	15 59.6	17 92.3	4 100.0			2	4
Multiple^*f*^ (13)						1 7.7	0 7.7	5 46.2	0 46.2	2 61.5	3 84.6	2 100.0			2	8
Carbapenemase negative (110)		1 0.9	1 1.8	5 6.4	11 16.4	12 27.3	7 33.6	20 51.8	19 69.1	17 84.5	10 93.6	5 98.2	0 98.2	2 100.0	0.5	4
*E. coli, K. pneumoniae*, and *P. mirabilis*																
Non-ESBL^*g*^ (8,538)	335 3.9	1,025 15.9	1,506 33.5	1,277 48.5	1,417 65.0	1,583 83.6	1,052 95.9	303 99.4	46 99.9	5 100.0					0.06	0.25
ESBL phenotype^*h*^ (2,179)	4 0.2	20 1.1	71 4.4	111 9.5	172 17.3	243 28.5	369 45.4	433 65.3	447 85.8	226 96.2	80 99.9	2 > 99.9	0 > 99.9	1 100.0	0.5	2
ESBL/AmpC^*i*^ (2,000)	3 0.2	15 0.9	50 3.4	89 7.9	150 15.4	224 26.6	348 44.0	409 64.4	418 85.3	215 96.0	76 99.8	2 99.9	0 99.9	1 100.0	0.5	2
ESBL only^*j*^ (1,825)	1 0.1	15 0.9	41 3.1	67 6.8	118 13.3	196 24.0	317 41.4	386 62.5	402 84.5	210 96.1	69 99.8	2 99.9	0 99.9	1 100.0	0.5	2
pAmpC only^*k*^ (131)	2 1.5	0 1.5	7 6.9	22 23.7	30 46.6	22 63.4	24 81.7	14 92.4	8 98.5	1 99.2	1 100.0				0.12	0.5
Multiple^*l*^ (44)			2 4.5	0 4.5	2 9.1	6 22.7	7 38.6	9 69.1	8 77.3	4 86.4	6 100.0				0.5	4

^*a*^ Enterobacterales susceptible to carbapenems were defined as isolates with meropenem and imipenem (excluding imipenem for *P. mirabilis*, *P. penneri*, and indole-positive Proteeae) MICs ≤ 1 mg/L; Enterobacterales classified as nonsusceptible were those with imipenem (excluding *P. mirabilis*, *P. penneri*, and indole-positive Proteeae) and/or meropenem MICs ≥ 2 mg/L. Organisms include *Citrobacter freundii* species complex (7), *C. koseri* (1), *E. cloacae* species complex (41), *Escherichia coli* (12), *Hafnia alvei* (1), *Klebsiella aerogenes* (27), *K. oxytoca* (11), *K. pneumoniae* (305), *Proteus mirabilis* (1), *Providencia rettgeri* (3), *Raoultella* species (5), *S. liquefaciens* complex (2), *S. marcescens* (25), and *Yokenella regensburgei* (1).

^*b*^ Includes a single *S. marcescens* with *bla*_SME-2_ excluded from further analysis.

^*c*^ Includes isolates carrying *bla*_KPC_ carbapenemase genes in the absence of MBL or *bla*_OXA-48-like_ genes.

^*d*^ Includes isolates carrying *bla*_OXA-48-like_ carbapenemase genes in the absence of MBL or *bla*_KPC_ genes.

^*e*^ Includes isolates carrying MBL carbapenemase genes in the absence of *bla*_OXA-48-like_ or *bla*_KPC_ genes.

^*f*^ Includes *K. pneumoniae* isolates carrying combinations of *bla*_VIM-1_+*bla*_KPC-3_ (4), *bla*_VIM-1_+*bla*_KPC-2_ (3), *bla*_NDM-1_+*bla*_OXA-48_ (3), *bla*_VIM-1_+*bla*_OXA-48_ (1), *bla*_NDM-1_+*bla*_OXA-181_ (1), and *bla*_KPC-2_+*bla*_NDM-1_ (1).

^*g*^ Includes carbapenem-susceptible *E. coli*, *K. pneumoniae*, and *P. mirabilis* that did not meet the CLSI MIC criteria (i.e., ceftriaxone, ceftazidime, and aztreonam MIC ≤1 mg/L) for screening of β-lactamases.

^*h*^ Includes carbapenem-susceptible *E. coli*, *K. pneumoniae*, and *P. mirabilis* that met the CLSI MIC criteria (i.e., either ceftriaxone, ceftazidime, or aztreonam MIC >1 mg/L) for screening of β-lactamases.

^*i*^ Includes carbapenem-susceptible *E. coli*, *K. pneumoniae*, and *P. mirabilis* that met the CLSI MIC criteria for screening of β-lactamases and carried ESBL and/or plasmidic AmpC genes. In addition, a total of 3 *E. coli* and 7 *K*. *pneumoniae* carried *bla*_OXA-48_-like; 1 *E. coli* and 2 *K*. *pneumoniae* carried *bla*_KPC_. Also, six *P. mirabilis* were positive for *bla*_VIM-4_ (1), *bla*_VIM-75_ (1), or *bla*_VIM-4_+*bla*_VIM-75_ (4).

^*j*^ Includes carbapenem-susceptible *E. coli*, *K. pneumoniae*, and *P. mirabilis* that met the CLSI MIC criteria for screening of β-lactamases and carried at least one ESBL-encoding gene and no plasmidic AmpC genes.

^*k*^ Includes carbapenem-susceptible *E. coli*, *K. pneumoniae*, and *P. mirabilis* that met the CLSI MIC criteria for screening of β-lactamases and carried *bla*_CMY_, *bla*_DHA_, or *bla*_FOX_ plasmidic AmpC genes without other acquired ESBL genes. One isolate carried *bla*_CMY_ and *bla*_DHA_ genes.

^*l*^ Includes carbapenem-susceptible *E. coli*, *K. pneumoniae,* and *P. mirabilis* that met the CLSI MIC criteria for screening of β-lactamases and possessed dual ESBL/pAmpC profiles. A single *E. coli* carried a *bla*_OXA-48_-like gene.

**TABLE 2:** Antimicrobial activity of cefiderocol tested against Enterobacterales subsets collected in 2020 and 2021 from US and European medical centers

Antimicrobial agent	MIC (mg/L)		CLSI^*a*^		EUCAST^*a*^		FDA^*a*^	
	50%/90%	Range	%S	%R	%S	%R	%S	%R
Carbapenem susceptible^*b*^ (15,623)								
Cefiderocol	0.06/0.5	0.004 to >64	99.9	0.1	99.2	0.8	99.9	0.1^*c*^
Imipenem-relebactam	0.12/0.5	0.03 to >8	95.1	0.8	99.2	0.8	95.1	0.8^*c*^
Meropenem-vaborbactam	0.03/0.06	0.015 to 1	100.0	0.0	100.0	0.0	100.0	0.0 ^*c*^
Ceftazidime-avibactam	0.12/0.25	0.015 to >32	99.9	0.1	99.9	0.1	99.9	0.1 ^*c*^
Piperacillin-tazobactam	2/16	0.06 to >128	86.9	8.9	86.9	13.1	86.9	8.9
Aztreonam	0.12/>16	0.03 to >16	83.2	14.9	81.1	16.8	83.2	14.9 ^*c*^
Ceftriaxone	0.06/>8	0.06 to >8	80.0	19.1	80.0	19.1 ^*d*^	80.0	19.1 ^*c*^
Ceftazidime	0.25/32	0.015 to >32	84.0	14.0	80.7	16.0	84.0	14.0 ^*c*^
Cefepime	0.06/16	0.03 to >32	86.4	11.1^*e*^	85.0	12.2	86.4	11.1^*c*^
Meropenem	0.03/0.06	0.015 to 1	100.0	0.0	100.0	0.0 ^*d*^	100.0	0.0 ^*c*^
Imipenem	0.12/1	0.12 to >8	94.0	0.9	99.1	0.1	94.0	0.9 ^*c*^
Ciprofloxacin	0.03/>4	0.008 to >4	77.5	19.1	77.5	19.1 ^*d*^	77.5	19.1 ^*c*^
Levofloxacin	0.06/8	0.015 to >32	80.2	16.5	80.2	16.5	80.2	16.5 ^*c*^
Amikacin	2/4	0.25 to >32	99.4	0.3	98.3	1.7 ^*f*^	99.4	0.3
Gentamicin	0.5/2	0.12 to >16	91.4	8.1	90.8	9.2 ^*f*^	91.4	8.1
Carbapenem nonsusceptible^*b*^ (442)								
Cefiderocol	1/4	0.008 to >64	96.6	0.7	85.1	14.9	96.6	0.7^*c*^
Imipenem-relebactam	0.5/>8	0.06 to >8	67.4	26.2	73.8	26.2	67.4	26.2^*c*^
Meropenem-vaborbactam	0.5/>8	0.015 to >8	74.7	21.9	78.1	21.9	74.7	21.9 ^*c*^
Ceftazidime-avibactam	1/>32	0.015 to >32	84.6	15.4	84.6	15.4	84.6	15.4 ^*c*^
Piperacillin-tazobactam	>128/>128	0.12 to >128	6.8	92.8	6.8	93.2	6.8	92.8
Aztreonam	>16/>16	0.03 to >16	17.0	82.1	15.2	83.0	17.0	82.1 ^*c*^
Ceftriaxone	>8/>8	0.06 to >8	11.3	87.8	11.3	87.8 ^*d*^	11.3	87.8 ^*c*^
Ceftazidime	>32/>32	0.03 to >32	14.9	83.7	12.4	85.1	14.9	83.7 ^*c*^
Cefepime	>32/>32	0.03 to >32	16.5	74.4^*e*^	14.0	79.6	16.5	74.4^*c*^
Meropenem	16/>32	0.03 to >32	16.5	74.0	26.0	52.9 ^*d*^	16.5	74.0 ^*c*^
Imipenem	8/>8	0.25 to >8	6.1	74.7	25.3	60.2	6.1	74.7 ^*c*^
Ciprofloxacin	>4/>4	0.008 to >4	19.0	78.7	19.0	78.7 ^*d*^	19.0	78.7 ^*c*^
Levofloxacin	16/>32	0.015 to >32	20.7	73.4	20.7	73.4	20.7	73.4 ^*c*^
Amikacin	8/>32	0.25 to >32	68.6	21.0	60.6	39.4 ^*f*^	68.6	21.0
Gentamicin	2/>16	0.12 to >16	54.8	39.3	50.9	49.1 ^*f*^	54.8	39.3
KPC (187)								
Cefiderocol	0.5/2	0.008 to 32	98.9	0.5	90.9	9.1	98.9	0.5 ^*c*^
Imipenem-relebactam	0.12/0.5	0.06 to 2	99.5 ^*b*^	0.0	100.0 ^*b*^	0.0	99.5	0.0 ^*c*^
Meropenem-vaborbactam	0.06/2	≤0.015 to >8	97.9	1.1	98.9	1.1	97.9	1.1 ^*c*^
Ceftazidime-avibactam	1/4	≤0.015 to >32	99.5	0.5	99.5	0.5	99.5	0.5 ^*c*^
Piperacillin-tazobactam	>128/>128	64 to >128	0.0	100.0	0.0	100.0	0.0	100.0
Aztreonam	>16/>16	16 to >16	0.0	100.0	0.0	100.0	0.0	100.0 ^*c*^
Ceftriaxone	>8/>8	8 to >8	0.0	100.0	0.0	100.0 ^*d*^	0.0	100.0 ^*c*^
Ceftazidime	>32/>32	2 to >32	2.7	96.8	0.0	97.3	2.7	96.8 ^*c*^
Cefepime	>32/>32	1 to >32	3.7	84.0^*e*^	0.5	91.4	3.7	84.0 ^*c*^
Meropenem	32/>32	0.5 to >32	2.7	89.3	10.7	65.8 ^*d*^	2.7	89.3 ^*c*^
Imipenem	>8/>8	2 to >8	0.0	97.3	2.7	86.1	0.0	97.3 ^*c*^
Ciprofloxacin	>4/>4	≤0.008 to >4	7.5	89.3	7.5	89.3 ^*d*^	7.5	89.3 ^*c*^
Levofloxacin	32/>32	≤0.015 to >32	10.2	84.0	10.2	84.0	10.2	84.0 ^*c*^
Amikacin	4/>32	0.5 to >32	50.3	41.7	58.3	41.7 ^*f*^	64.2	18.7
Gentamicin	4/>16	≤0.12 to >16	47.8	47.8	47.8	52.2 ^*f*^	52.2	38.2
MBL (52)								
Cefiderocol	2/4	0.06 to 8	92.3	0.0	59.6	40.4	92.3	0.0 ^*c*^
Imipenem-relebactam	>8/>8	2 to >8	0.0 ^*b*^	98.1	1.9 ^*b*^	98.1	0.0	98.1^*c*^
Meropenem-vaborbactam	>8/>8	0.5 to >8	17.3	75.0	25.0	75.0	17.3 ^*c*^	75.0 ^*c*^
Ceftazidime-avibactam	>32/>32	>32	0.0	100.0	0.0	100.0	0.0 ^*c*^	100.0 ^*c*^
Piperacillin-tazobactam	>128/>128	32 to >128	0.0	100.0	0.0	100.0	0.0	100.0
Aztreonam	>16/>16	≤0.03 to >16	25.0	69.2	23.1	75.0	25.0 ^*c*^	69.2 ^*c*^
Ceftriaxone	>8/>8	>8	0.0	100.0	0.0	100.0 ^*d*^	0.0 ^*c*^	100.0 ^*c*^
Ceftazidime	>32/>32	>32	0.0	100.0	0.0	100.0	0.0 ^*c*^	100.0 ^*c*^
Cefepime	>32/>32	8 to >32	0.0	98.1 ^*e*^	0.0	100.0	0.0	98.1 ^*c*^
Meropenem	32/>32	0.5 to >32	5.8	94.2	5.8	75.0 ^*d*^	5.8 ^*c*^	94.2 ^*c*^
Imipenem	>8/>8	4 to >8	0.0	100.0	0.0	80.8	0.0 ^*c*^	100.0 ^*c*^
Ciprofloxacin	>4/>4	0.015 to >4	15.4	82.7	15.4	82.7 ^*d*^	15.4 ^*c*^	82.7 ^*c*^
Levofloxacin	16/32	0.03 to >32	15.7	72.5	15.7	72.5	15.7 ^*c*^	72.5 ^*c*^
Amikacin	16/>32	1 to >32	21.2	57.7	42.3	57.7 ^*f*^	65.4	26.9
Gentamicin	8/>16	0.25 to >16	32.7	57.7	32.7	67.3 ^*f*^	42.3	50.0
OXA-48-like (79)								
Cefiderocol	0.5/2	0.008 to 4	100.0	0.0	92.4	7.6	100.0	0.0 ^*c*^
Imipenem-relebactam	4/>8	0.5 to >8	22.8 ^*b*^	60.8	39.2 ^*b*^	60.8	22.8	60.8^*c*^
Meropenem-vaborbactam	>8/>8	0.25 to >8	43.0	55.7	44.3	55.7	43.0	55.7^*c*^
Ceftazidime-avibactam	1/2	0.06 to 4	100.0	0.0	100.0	0.0	100.0	0.0 ^*c*^
Piperacillin-tazobactam	>128/>128	32 to >128	0.0	100.0	0.0	100.0	0.0	100.0
Aztreonam	>16/>16	0.06 to >16	31.6	68.4	29.1	68.4	31.6	68.4 ^*c*^
Ceftriaxone	>8/>8	0.12 to >8	25.3	70.9	25.3	70.9^*d*^	25.3	70.9 ^*c*^
Ceftazidime	>32/>32	0.25 to >32	30.4	67.1	26.6	69.6	30.4	67.1 ^*c*^
Cefepime	>32/>32	0.12 to >32	30.4	64.6 ^*e*^	26.6	67.1	30.4	64.6 ^*c*^
Meropenem	16/>32	0.5 to >32	27.8	60.8	39.2	55.7 ^*d*^	27.8	60.8 ^*c*^
Imipenem	4/>8	0.5 to >8	2.5	65.8	34.2	41.8	2.5	65.8 ^*c*^
Ciprofloxacin	>4/>4	≤0.008 to >4	13.9	83.5	13.9	83.5 ^*d*^	13.9	83.5 ^*c*^
Levofloxacin	16/>32	≤0.015 to >32	16.5	78.5	16.5	78.5	16.5	78.5 ^*c*^
Amikacin	16/>32	1 to >32	39.2	50.6	49.4	50.6 ^*f*^	55.7	41.8
Gentamicin	8/>16	0.25 to >16	48.1	51.9	48.1	51.9 ^*f*^	48.1	49.4
Multiple (13)								
Cefiderocol	2/8	0.12 to 8	84.6	0.0	61.5	38.5	84.6	0.0^*c*^
Imipenem-relebactam	>8/>8	4 to >8	0.0 ^*b*^	100.0	0.0 ^*b*^	100.0	0.0	100.0^*c*^
Meropenem-vaborbactam	>8/>8	4 to >8	7.7	76.9	23.1	76.9	7.7	76.9 ^*c*^
Ceftazidime-avibactam	>32/>32	>32	0.0	100.0	0.0	100.0	0.0	100.0 ^*c*^
Piperacillin-tazobactam	>128/>128	>128	0.0	100.0	0.0	100.0	0.0	100.0
Aztreonam	>16/>16	0.5 to >16	7.7	92.3	7.7	92.3	7.7	92.3 ^*c*^
Ceftriaxone	>8/>8	>8	0.0	100.0	0.0	100.0 ^*d*^	0.0	100.0 ^*c*^
Ceftazidime	>32/>32	>32	0.0	100.0	0.0	100.0	0.0	100.0 ^*c*^
Cefepime	>32/>32	32 to >32	0.0	100.0	0.0	100.0	0.0	100.0 ^*c*^
Meropenem	>32/>32	4 to >32	0.0	100.0	0.0	92.3 ^*d*^	0.0	100.0 ^*c*^
Imipenem	>8/>8	4 to >8	0.0	100.0	0.0	92.3	0.0	100.0 ^*c*^
Ciprofloxacin	>4/>4	>4	0.0	100.0	0.0	100.0 ^*d*^	0.0	100.0 ^*c*^
Levofloxacin	32/>32	4 to >32	0.0	100.0	0.0	100.0	0.0	100.0 ^*c*^
Amikacin	>32/>32	2 to >32	23.1	69.2	30.8	69.2 ^*f*^	30.8	53.8
Gentamicin	>16/>16	1 to >16	15.4	76.9	15.4	84.6 ^*f*^	23.1	76.9
Carbapenemase negative (110)								
Cefiderocol	0.5/4	0.008 to >64	93.6	1.8	84.5	15.5	93.6	1.8 ^*c*^
Imipenem-relebactam	0.25/2	0.06 to 8	85.5 ^*b*^	2.7	97.3 ^*b*^	2.7	85.5	2.7 ^*c*^
Meropenem-vaborbactam	0.5/4	0.03 to >8	92.7	1.8	98.2	1.8	92.7	1.8 ^*c*^
Ceftazidime-avibactam	1/4	0.03 to >32	98.2	1.8	98.2	1.8	98.2	1.8 ^*c*^
Piperacillin-tazobactam	>128/>128	0.12 to >128	26.4	71.8	26.4	73.6	26.4	71.8
Aztreonam	>16/>16	≤0.03 to >16	32.7	67.3	28.2	67.3	32.7	67.3 ^*c*^
Ceftriaxone	>8/>8	≤0.06 to >8	26.4	72.7	26.4	72.7 ^*d*^	26.4	72.7 ^*c*^
Ceftazidime	>32/>32	0.03 to >32	32.7	64.5	30.0	67.3	32.7	64.5 ^*c*^
Cefepime	16/>32	≤0.03 to >32	37.3	51.8 ^*e*^	35.5	57.3	37.3	51.8^*c*^
Meropenem	2/16	0.03 to >32	39.1	44.5	55.5	13.6 ^*d*^	39.1	44.5 ^*c*^
Imipenem	2/8	0.25 to >8	22.7	27.3	72.7	15.5	22.7	27.3 ^*c*^
Ciprofloxacin	1/>4	≤0.008 to >4	45.5	53.6	45.5	53.6 ^*d*^	45.5	53.6 ^*c*^
Levofloxacin	1/>32	≤0.015 to >32	45.9	49.5	45.9	49.5	45.9	49.5 ^*c*^
Amikacin	2/16	≤0.25 to >32	70.0	15.5	84.5	15.5 ^*f*^	90.9	3.6
Gentamicin	0.5/>16	≤0.12 to >16	70.6	26.6	70.6	29.4 ^*f*^	73.4	24.8

^*a*^ Criteria as published by CLSI (11), EUCAST (2023), and US FDA (2023). Analysis of imipenem-relebactam MIC using CLSI breakpoints excluded *Morganella* spp., *Proteus* spp., and *Providencia* spp., whereas EUCAST excluded *Morganellaceae*.

^*b*^ See footnotes on Table 1 for additional information.

^*c*^ Same breakpoints as those published by CLSI.

^*d*^ Using breakpoints for infections other than meningitis.

^*e*^ Percentage of intermediate results can be interpreted as susceptible-dose dependent.

^*f*^ For urinary tract infections, aminoglycosides must be used in combination with other active therapy for the treatment of systemic infections.

Cefiderocol (MIC_50/90_, 0.5/2 mg/L), imipenem-relebactam (MIC_50/90_, 0.12/0.5 mg/L), meropenem-vaborbactam (MIC_50/90_, 0.06/2 mg/L), and ceftazidime-avibactam (MIC_50/90_, 1/4 mg/L) were all active against the KPC subset (CLSI|FDA/EUCAST, 97.9%–99.5%/90.9%–100% susceptible) (Table 2). By contrast, cefiderocol (MIC_50/90_, 2/4 mg/L; CLSI|FDA/EUCAST, 92.3/59.6% susceptible) was the only agent active against Enterobacterales carrying MBL genes, whereas cefiderocol (MIC_50/90_, 0.5/2 mg/L; CLSI|FDA/EUCAST,100/92.4% susceptible) and ceftazidime-avibactam (1/2 mg/L; CLSI|FDA/EUCAST,100/100% susceptible) were the only active agents against isolates carrying *bla*_OXA-48_-like genes (Table 2). Cefiderocol (MIC_50/90_, 2/8 mg/L) inhibited at ≤4 mg/L 84.6% of the *K. pneumoniae* with dual carbapenemases. Against those carbapenemase-nonproducing, carbapenem-nonsusceptible isolates, cefiderocol inhibited 93.6%/84.5% (CLSI|FDA/EUCAST), similar to the activities of the β-lactam/β-lactamase inhibitor combinations tested (Table 2).

Among *Escherichia coli* (*n* = 6,883), *Klebsiella pneumoniae* (*n* = 2,902), and *Proteus mirabilis* (*n* = 933) susceptible to carbapenems, 20.3% (2,179/10,718) of these isolates displayed MIC values of ≥2 mg/L for aztreonam, ceftazidime, or ceftriaxone, the CLSI criteria for screening of β-lactamase genes (i.e., ESBL). ESBL and/or pAmpC genes were identified in 91.8% (2,000/2,179) of isolates screened (Table 1). The majority (91.3%; 1,825/2,000) of these isolates carried at least one ESBL-encoding gene, whereas small proportions of isolates carried pAmpC genes (6.6%; 131/2,000) or combinations of at least one gene from each group (2.2%; 44/2,000). Cefiderocol had MIC_50_ and MIC_90_ values of 0.5 mg/L and 2 mg/L, respectively, against the overall group of ESBL/AmpC and the ESBL-only subset of carbapenem-susceptible *E. coli*, *K. pneumoniae*, and *P. mirabilis* (Table 1). The cefiderocol MIC_50_ value of 0.12 mg/L and MIC_90_ value of 0.5 mg/L, noted against carbapenem-susceptible pAmpC producers, were fourfold lower than those values obtained against the groups described above (Table 1), whereas isolates with at least one ESBL and pAmpC gene displayed MIC_50/90_ values of 0.5 mg/L and 4 mg/L, respectively. Other antimicrobial agents tested against non-ESBL *E. coli*, *K. pneumoniae*, and *P. mirabilis* showed activity (≥93.5% susceptible), except for the fluoroquinolones (Table 3). By contrast, cefiderocol, ceftazidime-avibactam, the carbapenems, and the carbapenem combinations were active (≥96.1% susceptible) against carbapenem-susceptible, ESBL/pAmpC-producing *E. coli*, *K. pneumoniae*, and *P. mirabilis* (Table 3).

**TABLE 3:** Antimicrobial activity of cefiderocol tested against carbapenem-susceptible *E. coli, K. pneumoniae,* and *P. mirabilis* subsets collected in 2020 and 2021 from US and European medical centers

Antimicrobial agent			MIC (mg/L)					CLSI^*a*^			EUCAST^*a*^				FDA^*a*^	
			50%/90%		Range			%S		%R	%S		%R		%S	%R
Non-ESBL^*f*^ (8,538)																
Cefiderocol			0.06/0.25		≤0.004 to 2			100.0		0.0	100.0		0.0		100.0^*b*^	0.0
Imipenem-relebactam			0.12/0.5		≤0.03 to 8			93.6^*a*^		1.1	98.9^*a*^		1.1		93.6^*b*^	1.1
Meropenem-vaborbactam			0.03/0.06		≤0.015 to 1			100.0		0.0	100.0		0.0		100.0^*b*^	0.0
Ceftazidime-avibactam			0.12/0.25		≤0.015 to 1			100.0		0.0	100.0		0.0		100.0^*b*^	0.0
Piperacillin-tazobactam			2/4		≤0.06 to >128			95.5		2.5	95.5		4.5		95.5	2.5
Aztreonam			0.06/0.12		≤0.03 to 1			100.0		0.0	100.0		0.0		100.0^*b*^	0.0
Ceftriaxone			≤0.06/0.12		≤0.06 to 1			100.0		0.0	100.0^*c*^		0.0		100.0^*b*^	0.0
Ceftazidime			0.12/0.5		≤0.015 to 1			100.0		0.0	100.0		0.0		100.0^*b*^	0.0
Cefepime			0.06/0.12		≤0.03 to >32			99.7^*d*^		0.1	99.5		0.1		99.7^*b*^	0.1
Meropenem			≤0.015/0.03		≤0.015 to 1			100.0		0.0	100.0^*c*^		0.0		100.0^*b*^	0.0
Imipenem			0.12/0.5		≤0.12 to 8			93.6		0.8	99.2		<0.1		93.6^*b*^	0.8
Ciprofloxacin			0.03/>4		≤0.008 to >4			84.3		12.9	84.3^*c*^		12.9		84.3^*b*^	12.9
Levofloxacin			0.06/4		≤0.015 to >32			86.2		11.6	86.2		11.6		86.2^*b*^	11.6
Amikacin			2/4		≤0.25 to >32			93.5		0.6	99.4^*e*^		0.6		99.9	<0.1
Gentamicin			0.5/1		≤0.12 to >16			94.7		4.9	94.7^*e*^		5.3		95.1	4.7
ESBL^*f*^ (1,825)																
Cefiderocol			0.5/2		≤0.004 to >64			99.8		0.1	96.1		3.9		99.8^*b*^	0.1
Imipenem-relebactam			0.12/0.25		≤0.03 to >8			98.1^*a*^		0.9	99.1^*a*^		0.9		98.1^*b*^	0.9
Meropenem-vaborbactam			0.03/0.03		≤0.015 to 1			100.0		0.0	100.0		0.0		100.0^*b*^	0.0
Ceftazidime-avibactam			0.12/0.5		≤0.015 to >32			99.6		0.4	99.6		0.4		99.6^*b*^	0.4
Piperacillin-tazobactam			4/64		0.12 to >128			68.6		18.7	68.6		31.4		68.6	18.7
Aztreonam			>16/>16		0.06 to >16			8.3		82.0	3.2		91.7		8.3^*b*^	82.0
Ceftriaxone			>8/>8		≤0.06 to >8			0.4		99.2	0.4^*c*^		99.2		0.4^*b*^	99.2
Ceftazidime			32/>32		0.06 to >32			19.0		69.6	4.7		81.0		19.0^*b*^	69.6
Cefepime			>32/>32		0.12 to >32			4.5^*d*^		86.0	2.2		91.7		4.5^*b*^	86.0
Meropenem			0.03/0.06		≤0.015 to 1			100.0		0.0	100.0^*c*^		0.0		100.0^*b*^	0.0
Imipenem			≤0.12/0.5		≤0.12 to >8			98.1		0.8	99.2		0.4		98.1^*b*^	0.8
Ciprofloxacin			>4/>4		≤0.008 to >4			14.6		76.8	14.6^*c*^		76.8		14.6^*b*^	76.8
Levofloxacin			8/32		≤0.015 to >32			24.5		66.1	24.5		66.1		24.5^*b*^	66.1
Amikacin			4/8		≤0.25 to >32			75.1		9.0	91.0^*e*^		9.0		96.7	1.5
Gentamicin			1/>16		≤0.12 to >16			61.1		38.4	61.1^*e*^		38.9		61.6	37.2
pAmpC (131)																
Cefiderocol			0.12/0.5		≤0.004 to 4			100.0		0.0	99.2		0.8		100.0^*b*^	0.0
Imipenem-relebactam			0.12/2		≤0.03 to 4			84.7^*a*^		2.3	97.7^*a*^		2.3		84.7^*b*^	2.3
Meropenem-vaborbactam			0.03/0.06		≤0.015 to 0.12			100.0		0.0	100.0		0.0		100.0^*b*^	0.0
Ceftazidime-avibactam			0.12/0.5		≤0.015 to 2			100.0		0.0	100.0		0.0		100.0^*b*^	0.0
Piperacillin-tazobactam			4/32		0.5 to >128			75.6		10.7	75.6		24.4		75.6	10.7
Aztreonam			4/>16		0.12 to >16			53.4		22.9	25.2		46.6		53.4^*b*^	22.9
Ceftriaxone			>8/>8		0.25 to >8			19.1		70.2	19.1^*c*^		70.2		19.1^*b*^	70.2
Ceftazidime			32/>32		0.5 to >32			14.5		74.0	0.8		85.5		14.5^*b*^	74.0
Cefepime			0.25/2		≤0.03 to 8			93.9^*d*^		0.0	88.5		0.8		93.9^*b*^	0.0
Meropenem			0.03/0.12		≤0.015 to 0.5			100.0		0.0	100.0^*c*^		0.0		100.0^*b*^	0.0
Imipenem			0.5/2		≤0.12 to 4			81.7		9.9	90.1		0.0		81.7 ^*b*^	9.9
Ciprofloxacin			0.25/>4		≤0.008 to >4			51.9		39.7	51.9^*c*^		39.7		51.9^*b*^	39.7
Levofloxacin			0.5/32		≤0.015 to >32			57.3		34.4	57.3		34.4		57.3^*b*^	34.4
Amikacin			4/8		0.5 to >32			84.0		6.1	93.9^*e*^		6.1		94.7	5.3
Gentamicin			1/16		≤0.12 to >16			84.7		15.3	84.7^*e*^		15.3		84.7	14.5
ESBL+ pAmpC (44)																
Cefiderocol			0.5/4		0.015 to 4			100.0		0.0	86.4		13.6		100.0^*b*^	0.0
Imipenem-relebactam			0.12/0.25		≤0.03 to 1			100.0^*a*^		0.0	100.0^*a*^		0.0		100.0^*b*^	0.0
Meropenem-vaborbactam			0.03/0.06		≤0.015 to 0.5			100.0		0.0	100.0		0.0		100.0^*b*^	0.0
Ceftazidime-avibactam			0.12/2		≤0.015 to 4			100.0		0.0	100.0		0.0		100.0^*b*^	0.0
Piperacillin-tazobactam			16/>128		0.5 to >128			47.7		45.5	47.7		52.3		47.7	45.5
Aztreonam			>16/>16		0.5 to >16			13.6		79.5	9.1		86.4		13.6^*b*^	79.5
Ceftriaxone			>8/>8		≤0.06 to >8			11.4		86.4	11.4^*c*^		86.4		11.4^*b*^	86.4
Ceftazidime			>32/>32		4 to >32			6.8		86.4	0.0		93.2		6.8^*b*^	86.4
Cefepime			>32/>32		≤0.03 to >32			25.0^*d*^		63.6	20.5		72.7		25.0^*b*^	63.6
Meropenem			0.03/0.12		≤0.015 to 1			100.0		0.0	100.0^*c*^		0.0		100.0^*b*^	0.0
Imipenem			0.25/1		≤0.12 to 1			100.0		0.0	100.0		0.0		100.0^*b*^	0.0
Ciprofloxacin			>4/>4		0.25 to >4			4.5		86.4	4.5^*c*^		86.4		4.5^*b*^	86.4
Levofloxacin			16/>32		0.25 to >32			15.9		72.7	15.9		72.7		15.9^*b*^	72.7
Amikacin			4/8		0.5 to >32			70.5		9.1	90.9^*e*^		9.1		95.5	2.3
Gentamicin			1/>16		0.25 to >16			61.4		38.6	61.4^*e*^		38.6		61.4	31.8

^*a*^ Criteria as published by CLSI (2023), EUCAST (2023), and US FDA (2023). Analysis of imipenem-relebactam MIC using CLSI breakpoints excluded *Morganella* spp., *Proteus* spp., and *Providencia* spp., whereas EUCAST excluded *Morganellaceae*.

^*b*^ Same breakpoints as those published by CLSI.

^*c*^ Using breakpoints for infections other than meningitis.

^*d*^ Percentage of intermediate results can be interpreted as susceptible-dose dependent.

^*e*^ For urinary tract infections, aminoglycosides must be used in combination with other active therapy for the treatment of systemic infections.

^*f*^ See footnotes on Table 1 for additional information.

In all, 30 (0.2%) Enterobacterales were nonsusceptible to cefiderocol per CLSI criteria (MIC, ≥8 mg/L; Table S3). These isolates were comprised primarily of *Enterobacter* spp. (*n* = 12) and *K. pneumoniae* (*n* = 10), with the remaining eight isolates represented by four species groups. No single feature united these isolates, as no sequence type dominated any organism group and isolates harbored numerous combinations of intrinsic β-lactamases, alterations therein, and acquired resistance mechanisms including ESBLs and/or pAmpCs (*n* = 21) and carbapenemases (*n* = 9).

### P. aeruginosa

A total of 4,681 *P*. *aeruginosa* isolates were included in this study and a breakdown of the infection type and geographic distribution of the *P. aeruginosa* isolates collected for this study is displayed in Tables S1 and S2. Among the 4,681 *P*. *aeruginosa*, 16.9% (793/4,681) and 4.3% (200/4,681) showed an MDR or DTR phenotype, respectively. Nonsusceptibility to cephalosporins (ceftazidime and/or cefepime) and/or carbapenem (imipenem and/or meropenem) was observed in 33.3% (1,559/4,681) of isolates, which were then screened for acquired β-lactamase genes (Table 4). Only a small number of these isolates (76; 4.9%) carried carbapenemase genes, most of which (73.7%) encoded at least one class B carbapenemase. Class A carbapenemase genes were identified in 21 isolates with profiles comprised of *bla*_GES-5_ (16 strains; one strain *bla*_GES-5_+*bla*_VIM-2_), *bla*_GES-6_ (four strains), or *bla*_KPC-2_ (one strain) (Table 4). Most carbapenemase producers (90.8%; 69/76) originated from Europe (data not shown). Cefiderocol had similar MIC values against cephalosporin- and carbapenem-susceptible *P. aeruginosa* (MIC_50/90_, 0.06/0.25 mg/L) and cephalosporin- and/or carbapenem-non-susceptible *P. aeruginosa* (MIC_50/90_, 0.12/0.5 mg/L), as well as against the MDR and DTR subsets (MIC_50/90_, 0.12/1 mg/L). High susceptibilities (≥97.8%) were obtained for all agents when tested against cephalosporin- and carbapenem-susceptible *P. aeruginosa* (Table 5). Cefiderocol (CLSI/EUCAST/FDA, 99.3/98.5/96.5% susceptible), imipenem-relebactam (87.8% susceptible), ceftazidime-avibactam (88.8% susceptible), and ceftolozane-tazobactam (88.1% susceptible) were the most active agents tested against cephalosporin- and/or carbapenem-non-susceptible *P. aeruginosa* (Table 5). Cefiderocol also had the greatest activity against the MDR (CLSI/EUCAST/FDA, 98.6/97.4/94.7% susceptible) and DTR (CLSI/EUCAST/FDA, 97.5/96.5/90.0% susceptible) subsets of *P. aeruginosa*, whereas other agents tested showed susceptibility results < 80%, except for colistin (99.5% susceptible) (Table 5).

**TABLE 4:** MIC distribution of cefiderocol obtained against phenotypic and genotypic resistance subsets of *P. aeruginosa* collected in 2020 and 2021 from US and European medical centers

Organism/group (no. of isolates)	No. and cumulative % of isolates inhibited at MIC (mg/L) of:														MIC_50_	MIC_90_
	≤0.004	0.008	0.015	0.03	0.06	0.12	0.25	0.5	1	2	4	8	16	>16		
*P. aeruginosa* (4,681)	124 2.6	85 4.5	191 8.5	611 21.6	1,232 47.9	1,350 76.8	691 91.5	231 96.5	101 98.6	39 99.4	14 99.7	6 99.9	4 > 99.9	2 100.0	0.12	0.25
Cephalosporin/carbapenem-S (3,122)^*a*^	92 2.9	58 4.8	135 9.1	474 24.3	878 52.4	905 81.4	407 94.5	122 98.4	40 99.6	9 99.9	1 > 99.9	1 100.0			0.06	0.25
Cephalosporin/carbapenem-NS (1,559)^*b*^	32 2.1	27 3.8	56 7.4	137 16.2	354 38.9	445 67.4	284 85.6	109 92.6	61 96.5	30 98.5	13 99.3	5 99.6	4 99.9	2 100	0.12	0.5
MDR (793)^*c*^	13 1.6	10 2.9	23 5.8	56 12.9	163 33.4	214 60.4	168 81.6	66 89.9	38 94.7	21 97.4	10 98.6	5 99.2	4 99.7	2 100	0.12	1
DTR (200)^*c*^	4 2.0	3 3.5	2 4.5	11 10.0	36 28.0	63 59.5	34 76.5	15 84.0	12 90.0	13 96.5	2 97.5	3 99.0	1 99.5	1 100	0.12	1
Carbapenemase positive (76)^*d*^	1 1.3	0 1.3	0 1.3	3 5.3	12 21.3	28 58.7	11 73.3	5 80.0	6 88.0	6 96.0	2 98.7	1 100.0			0.12	2
MBL (56)^*e*^	1 1.8	0 1.8	1 3.6	3 8.9	6 19.6	16 48.2	9 64.3	5 73.2	6 83.9	6 94.6	2 98.2	1 100.0			0.25	2
VIM (43)^*f*^			1 2.3	3 9.3	5 20.9	15 55.8	8 74.4	3 81.4	3 88.4	4 97.7	2 100.0				0.12	2
Other MBL (14)^*g*^	1 7.1	0 7.1	0 7.1	0 7.1	1 14.3	1 21.4	1 28.6	2 42.9	3 643	2 78.6	2 92.9	1 100.0			1	4
Serine carbapenemase (21)^*h*^					7 33.3	12 90.5	2 100.0								0.12	0.12
Carbapenemase negative (1,483)	31 2.1	27 3.9	55 7.6	134 16.7	342 39.7	417 67.8	273 86.2	104 93.3	55 97.0	24 98.6	11 99.3	4 99.6	4 99.9	2 100.0	0.12	0.5

^*a*^ Includes isolates with imipenem and meropenem MIC values ≤ 2 mg/L and ceftazidime and cefepime MIC ≤8 mg/L.

^*b*^ Includes isolates with imipenem and/or meropenem MIC values ≥ 4 mg/L and/or ceftazidime and/or cefepime MICs ≥ 16 mg/L.

^*c*^ MDR, multi-drug-resistant isolate; DTR, difficult-to-treat resistance (see Materials and Methods for resistance definitions).

^*d*^ Includes genes encoding FIM-1 (1), VIM-4 (4), GES-5 (15), GES-6 (4), IMP-1 (1), IMP-7 (3), IMP-13 (4), KPC-2 (1), NDM-1 (4), VIM-1 +HMB-1 (1), VIM-1 (5), VIM-2 + GES-5 (1), VIM-2 (30), VIM-20 (1), VIM-43 (1).

^*e*^ Includes genes encoding FIM-1 (1), VIM-4 (4), IMP-1 (1), IMP-7 (3), IMP-13 (4), NDM-1 (4), VIM-1 + HMB-1 (1), VIM-1 (5), VIM-2 + GES-5 (1), VIM-2 (30), VIM-20 (1), VIM-43 (1).

^*f*^ Includes genes encoding VIM-4 (4), VIM-1 + HMB-1 (1), VIM-1 (5), VIM-2 + GES-5 (1), VIM-2 (30), VIM-20 (1), VIM-43 (1).

^*g*^ Includes genes encoding FIM-1 (1), IMP-1 (1), IMP-7 (3), IMP-13 (4), NDM-1 (4), VIM-1 + HMB-1 (1).

^*h*^ Includes genes encoding GES-5 (15), GES-6 (4), KPC-2 (1), VIM-2 + GES-5 (1).

**TABLE 5:** Antimicrobial activity of cefiderocol and comparator agents tested against *P. aeruginosa* subsets collected in 2020 and 2021 from US and European medical centers

Organism group Antimicrobial agent	MIC (mg/L)		CLSI^*a*^		EUCAST^*a*^		FDA^*a*^	
	50%/90%	Range	%S	%R	%S	%R	%S	%R
Cephalosporin/carbapenem-S^*b*^ (3,122)								
Cefiderocol	0.06/0.25	≤0.004 to 8	>99.9	0.0	99.9	0.1	99.6	0.1
Imipenem-relebactam	0.25/0.25	≤0.03 to 2	100.0	0.0	100.0	0.0	100.0^*c*^	0.0
Meropenem-vaborbactam	0.25/1	≤0.015 to 4			100.0	0.0		
Ceftazidime-avibactam	2/2	≤0.015 to 16	>99.9	<0.1	>99.9	<0.1	>99.9^*c*^	<0.1
Ceftolozane-tazobactam	0.5/1	≤0.12 to 8	>99.9	0.0	>99.9	<0.1	>99.9^*c*^	0.0
Ceftazidime	2/4	0.06 to 8	100.0	0.0	100.0^*d*^	0.0	100.0	0.0
Cefepime	2/4	0.06 to 8	100.0	0.0	100.0^*d*^	0.0	100.0	0.0
Piperacillin-tazobactam	4/8	≤0.06 to 128	97.8	0.3	100.0^*d*^	2.2	90.3	2.2
Meropenem	0.25/1	≤0.015 to 2	100.0	0.0	100.0^*e*^	0.0	100.0^*c*^	0.0
Colistin	1/1	≤0.06 to >8		0.4	99.8	0.2		
Cephalosporin/carbapenem-NS^*f*^ (1,559)								
Cefiderocol	0.12/0.5	≤0.004 to >64	99.3	0.4	98.5	1.5	96.5	1.5
Imipenem-relebactam	1/4	≤0.03 to >8	87.8	6.5	87.8	12.2	87.8^*c*^	6.5
Meropenem-vaborbactam	4/>8	≤0.015 to >8			70.9	29.1		
Ceftazidime-avibactam	4/16	0.12 to >32	88.8	11.2	88.8	11.2	88.8^*c*^	11.2
Ceftolozane-tazobactam	1/8	≤0.12 to >16	88.1	7.8	88.1	11.9	88.1^*c*^	7.8
Ceftazidime	16/>32	0.25 to >32	46.8	40.5	46.8^*d*^	53.2	46.8	53.2
Cefepime	8/32	0.25 to >32	56.1	16.4	56.1^*d*^	43.9	56.1	43.9
Piperacillin-tazobactam	32/>128	0.12 to >128	40.3	45.4	40.3^*d*^	59.7	25.7	59.7
Meropenem	4/32	0.03 to >32	36.4	46.5	36.4^*e*^	28.0	36.4^*c*^	46.5
Colistin	1/1	≤0.06 to >8		0.4	99.8	0.2		
MDR^*g*^ (793)								
Cefiderocol	0.12/1	≤0.004 to >64	98.6	0.8	97.4	2.6	94.7	2.6
Imipenem-relebactam	1/8	≤0.03 to >8	76.5	12.5	76.5	23.5	76.5^*c*^	12.5
Meropenem-vaborbactam	8/>8	0.03 to >8			52.3	47.7		
Ceftazidime-avibactam	4/32	0.5 to >32	78.4	21.6	78.4	21.6	78.4^*c*^	21.6
Ceftolozane-tazobactam	2/>16	0.25 to >16	77.5	14.9	77.5	22.5	77.5^*c*^	14.9
Ceftazidime	32/>32	1 to >32	25.0	69.1	25.0^*d*^	75.0	25.0	75.0
Cefepime	16/>32	2 to >32	29.6	29.8	29.6^*d*^	70.4	29.6	70.4
Piperacillin-tazobactam	128/>128	0.5 to >128	11.1	66.5	11.1^*d*^	88.9	4.4	88.9
Meropenem	8/32	0.06 to >32	18.0	70.0	18.0^*e*^	46.9	18.0^*c*^	70.0
Colistin	1/1	≤0.06 to >8		0.8	99.5	0.5		
DTR^*g*^ (200)								
Cefiderocol	0.12/1	≤0.004 to >64	97.5	1.0	96.5	3.5	90.0	3.5
Imipenem-relebactam	2/>8	0.5 to >8	56.5	26.0	56.5	43.5	56.5^*c*^	26.0
Meropenem-vaborbactam	>8/>8	1 to >8			27.5	72.5		
Ceftazidime-avibactam	8/>32	1 to >32	57.0	43.0	57.0	43.0	57.0^*c*^	43.0
Ceftolozane-tazobactam	4/>16	1 to >16	55.5	29.5	55.5	44.5	55.5^*c*^	29.5
Ceftazidime	>32/>32	16 to >32	0.0	84.0	0.0^*d*^	100.0	0.0	100.0
Cefepime	32/>32	16 to >32	0.0	57.5	0.0^*d*^	100.0	0.0	100.0
Piperacillin-tazobactam	128/>128	32 to >128	0.0	88.0	0.0^*d*^	100.0	0.0	100.0
Meropenem	16/>32	4 to >32	0.0	93.0	0.0^*e*^	92.1	0.0^*c*^	93.0
Colistin	1/1	0.12 to >8		0.5	99.5	0.5		
Carbapenemase positive (76)								
Cefiderocol	0.12/2	≤0.004 to 8	98.7	0.0	96.1	3.9	88.2	3.9
Imipenem-relebactam	>8/>8	0.25 to >8	3.9	94.7	3.9	96.1	3.9^*c*^	94.7
Meropenem-vaborbactam	>8/>8	0.25 to >8			9.2	90.8		
Ceftazidime-avibactam	32/>32	2 to >32	28.9	71.1	28.9	71.1	28.9^*c*^	71.1
Ceftolozane-tazobactam	>16/>16	1 to >16	1.3	86.8	1.3	98.7	1.3^*c*^	86.8
Ceftazidime	>32/>32	8 to >32	3.9	82.9	3.9^*d*^	96.1	3.9	96.1
Cefepime	32/>32	2 to >32	14.5	53.9	14.5^*d*^	85.5	14.5	85.5
Piperacillin-tazobactam	64/>128	8 to >128	3.9	76.3	3.9^*d*^	96.1	1.3	96.1
Meropenem	>32/>32	0.5 to >32	1.3	96.1	1.3^*e*^	92.1	1.3^*c*^	96.1
Colistin	1/1	0.25 to 2		0.0	100.0	0.0		
VIM-type (43)								
Cefiderocol	0.12/2	0.015 to 4	100.0	0.0	97.7	2.3	88.4	2.3
Imipenem-relebactam	>8/>8	4 to >8	0.0	97.7	0.0	100.0	0.0^*c*^	97.7
Meropenem-vaborbactam	>8/>8	4 to >8			11.6	88.4		
Ceftazidime-avibactam	32/>32	2 to >32	7.0	93.0	7.0	93.0	7.0^*c*^	93.0
Ceftolozane-tazobactam	>16/>16	16 to >16	0.0	100.0	0.0	100.0	0.0^*c*^	100.0
Ceftazidime	32/>32	8 to >32	4.7	86.0	4.7^*d*^	95.3	4.7	95.3
Cefepime	32/>32	2 to >32	7.0	62.8	7.0^*d*^	93.0	7.0	93.0
Piperacillin-tazobactam	64/>128	8 to >128	2.3	74.4	2.3^*d*^	97.7	2.3	97.7
Meropenem	>32/>32	4 to >32	0.0	95.3	0.0^*d*^	88.4	0.0^*c*^	95.3
Colistin	1/1	0.5 to 2		0.0	100.0	0.0		
Carbapenemase negative (1,483)								
Cefiderocol	0.12/0.5	≤0.004 to >64	99.3	0.4	98.6	1.4	97.0	1.4
Imipenem-relebactam	1/2	≤0.03 to >8	92.1	2.0	92.1	7.9	92.1^*c*^	2.0
Meropenem-vaborbactam	4/>8	≤0.015 to >8			74.1	25.9		
Ceftazidime-avibactam	4/8	0.12 to >32	91.8	8.2	91.8	8.2	91.8^*c*^	8.2
Ceftolozane-tazobactam	1/4	≤0.12 to >16	92.5	3.7	92.5	7.5	92.5^*c*^	3.7
Ceftazidime	16/>32	0.25 to >32	49.0	38.3	48.0^*d*^	51.0	49.0	51.0
Cefepime	8/32	0.25 to >32	58.2	14.5	58.2^*d*^	41.8	58.2	41.8
Piperacillin-tazobactam	32/>128	0.12 to >128	42.2	43.9	42.2^*d*^	57.8	26.9	57.8
Meropenem	4/16	0.03 to >32	38.2	44.0	38.2^*e*^	24.7	38.2^*c*^	44.0
Colistin	1/1	≤0.06 to >8		0.4	99.8	0.2		

^*a*^ Criteria as published by CLSI (2023), EUCAST (2023), and US FDA (2023).

^*b*^ Ceftazidime and cefepime MIC <16 mg/L and imipenem and meropenem MIC <4 mg/L.

^*c*^ Same breakpoints as those published by CLSI.

^*d*^ Results can be considered as susceptible only when treating infections where an increased concentration can be achieved by increased dosing regimens or increased concentration in the site of infections.

^*e*^ Using EUCAST breakpoints for infections other than meningitis.

^*f*^ Ceftazidime and/or cefepime MIC ≥16 mg/L and imipenem and/or meropenem MIC ≥4 mg/L.

^*g*^ MDR, multi-drug resistant; DTR, difficult-to-treat resistance. See Materials and Methods for definitions.

Against cephalosporin- and/or carbapenem-nonsusceptible isolates with carbapenemase genes, cefiderocol (MIC_50/90_, 0.12/2 mg/L) inhibited 98.7/96.1/88.2% of isolates according to CLSI, EUCAST, and FDA breakpoints, respectively (Tables 4 and 5). Furthermore, cefiderocol demonstrated comparable activity (MIC_50/90_, 0.12/2 mg/L; 100.0/97.7/88.4% susceptible per CLSI/EUCAST/FDA) against those *P. aeruginosa* possessing VIM-like carbapenemase genes, the most common carbapenemase type detected (*n* = 43; Table 5), whereas comparator agents demonstrated poor activity (<50% susceptible), including the β-lactam/β-lactamase inhibitor combinations imipenem-relebactam (0.0% susceptible), ceftazidime-avibactam (7.0%), and ceftolozane-tazobactam (0.0%). By contrast, cefiderocol (97.0%–99.3% susceptible) and the β-lactam/β-lactamase inhibitor combinations imipenem-relebactam (92.1% susceptible), ceftazidime-avibactam (91.8%), and ceftolozane-tazobactam (92.5%) were active against cephalosporin- and/or carbapenem-non-susceptible *P. aeruginosa* that did not carry carbapenemases (Table 5).

Twelve isolates produced cefiderocol MIC values > 4 mg/L (CLSI nonsusceptible breakpoint), and MIC values ranged from 8 to >64 mg/L (Table 4; Table S4). A single isolate bore an acquired carbapenemase gene (*bla*_NDM-1_; MIC, 8 mg/L), and six of the isolates contained alterations in either the Ω-loop (*n =* 6) or R2-loop (*n* = 2) region of PDC. The lack of acquired β-lactamase genes known to affect cefiderocol susceptibility suggests the intrinsic mechanisms of resistance influence this phenotype in a similar manner to evolved β-lactam and/or carbapenem resistance in *P. aeruginosa*. Most cefiderocol nonsusceptible isolates were derived from unique sequence types and no single molecular feature obviously united these isolates (Table S4).

### *Acinetobacter* spp.

*Acinetobacter* species included in this study were comprised primarily of *A. baumannii-calcoaceticus* species complex (1,443/1,613; 89.5%) (Table 6). A breakdown of the major species groupings by infection type and geographic source is displayed in Tables S1 and S2. Among all included isolates, 57.5% (927/1,613) were nonsusceptible to cephalosporins (ceftazidime and/or cefepime) and/or carbapenems (imipenem and/or meropenem), and *A. baumannii-calcoaceticus* species complex isolates accounted for 95.8% (888/927) of all isolates examined (data not shown). Among these 888 *A*. *baumannii-calcoaceticus* species complex isolates screened for β-lactamase genes, acquired carbapenemase and/or ESBL genes were detected in 87.6% (778/888) of isolates. The vast majority of carbapenemase genes belonged to class D β-lactamases (Table 6). Cefiderocol demonstrated MIC_50_ results of 0.06 mg/L and MIC_90_ values of 0.5 mg/L when tested against cephalosporin- and/or carbapenem-susceptible *Acinetobacter* spp. (Table 6). MIC_90_ values of 2 mg/L were obtained for cefiderocol against cephalosporin- and/or carbapenem-nonsusceptible *Acinetobacter* spp. and each subset, except against those carrying *bla*_OXA-24_-like or non-*bla*_OXA-23/24_-like genes (MIC_90_, 1 mg/L; Table 6). High susceptibility results (95.1%–100%) were obtained for all agents tested against cephalosporin- and/or carbapenem-susceptible *Acinetobacter* spp. for which breakpoints are available (Table 7). By contrast, only cefiderocol (MIC_50/90_, 0.12–0.25/1-2 mg/L) was active against cephalosporin- and/or carbapenem-nonsusceptible *Acinetobacter* spp. and against the additional resistance subsets (Table 7).

**TABLE 6:** MIC distribution of cefiderocol obtained against phenotypic and genotypic resistance subsets of *Acinetobacter* spp. collected in 2020 and 2021 from US and European medical centers

Organism/group (no. of isolates)	No. and cumulative % of isolates inhibited at MIC (mg/L) of:														MIC_50_	MIC_90_
	≤0.004	0.008	0.015	0.03	0.06	0.12	0.25	0.5	1	2	4	8	16	>16		
*Acinetobacter* spp. (1,613)	1 0.1	3 0.3	63 4.2	187 15.7	297 34.2	314 53.6	274 70.6	241 85.6	123 93.2	57 96.7	26 98.3	10 98.9	8 99.4	9 100.0	0.12	1
*A. baumannii-calcoaceticus* (1,443)		3 0.2	43 3.2	149 13.5	250 30.8	285 50.6	263 68.8	227 84.5	117 92.7	54 96.4	25 98.1	10 98.8	8 99.4	9 100.0	0.12	1
Other *Acinetobacter* spp. (170)^*a*^	1 0.6	0 0.6	20 12.4	38 34.7	47 62.4	29 79.4	11 85.9	14 94.1	6 97.6	3 99.4	1 100.0				0.06	0.5
Cephalosporin/carbapenem-S (686)^*b*^	1 0.1	3 0.3	57 8.9	167 33.2	208 63.6	120 81.0	61 89.9	41 95.9	20 98.8	4 99.4	2 99.7	2 100.0			0.06	0.5
Cephalosporin/carbapenem-NS (927)^*c*^			6 0.6	20 2.8	89 12.4	194 33.3	213 56.3	200 77.9	103 89.0	53 94.7	24 97.3	8 98.2	8 99.0	9 100.0	0.25	2
MDR (847)^*d*^			3 0.4	11 1.7	63 9.1	183 30.7	199 54.2	194 77.1	97 88.5	48 94.2	24 97.0	8 98.0	8 98.9	9 100.0	0.25	2
Carbapenemase (778)			2 0.3	6 1.0	63 9.1	170 31.0	180 54.1	182 77.5	90 89.1	43 94.6	22 97.4	7 98.3	7 99.2	6 100.0	0.25	2
OXA-23-group^*e*^ (588)			1 0.2	3 0.7	45 8.3	131 30.6	138 54.1	132 76.5	70 88.4	32 93.9	18 96.9	6 98.0	6 99.0	6 100.0	0.25	2
OXA-24-group^*f*^ (166)			1 0.6	1 1.2	13 9.0	31 27.7	38 50.6	50 80.7	17 91.0	11 97.6	3 99.4	0 99.4	1 100.0		0.25	1
Other OXA^*g*^ (30)			1 3.3	2 10	6 30.0	6 50.0	6 70.0	4 83.3	3 93.3	1 96.7	1 100.0				0.12	1
No carbapenemases^*h*^ (110)			1 0.9	6 6.4	13 18.2	18 34.5	30 61.8	14 74.5	12 85.5	9 93.6	2 95.5	1 96.4	1 97.3	3 100.0	0.25	2

^*a*^ Includes *Acinetobacter beijerinckii* (1), *A. bereziniae* (19), *A. courvalinii* (6), *A. dispersus* (1), *A. gerneri* (1), *A. guillouiae* (2), *A. gyllenbergii* (2), *A. haemolyticus* (2), *A. johnsonii* (12), *A. junii* (20), *A. lwoffii* (10), *A. proteolyticus* (6), *A. radioresistens* (26), *A. schindleri* (3), *A. soli* (3), *A. ursingii* (40), *A. variabilis* (2), *A. vivianii* (3), and *Acinetobacter* spp (11). .

^*b*^ Includes isolates with imipenem and meropenem MIC values ≤ 2 mg/L and ceftazidime and cefepime MIC ≤8 mg/L.

^*c*^ Includes isolates with imipenem and/or meropenem MIC values ≥ 4 mg/L and/or ceftazidime and/or cefepime MICs ≥ 16 mg/L.

^*d*^ MDR, multi-drug resistant, see Materials and Methods for definitions.

^*e*^ Includes isolates in which *bla*_OXA-23-like_ genes were detected, excluding isolates in which *bla*_OXA-24-like_ genes were also identified.

^*f*^ Includes isolates in which *bla*_OXA-24-like_ genes were detected, excluding isolates in which *bla*_OXA-23-like_ genes were also identified.

^*g*^ Includes isolates in which *bla*_OXA-like_ carbapenemase genes were detected, excluding isolates in which *bla*_OXA-23-like_, *bla*_OXA-24-like_, or *bla*_OXA-51-like_ were also identified.

^*h*^ Includes molecularly characterized isolates in which acquired carbapenemase genes were not detected.

**TABLE 7:** Antimicrobial activity of cefiderocol and comparator agents tested against *A. baumannii* subsets collected in 2020 and 2021 from US and European medical centers

Organism group Antimicrobial agent	MIC (mg/L)		CLSI^*a*^		EUCAST^*a*^		FDA^*a*^	
	50%/90%	Range	%S	%R	%S	%R	%S	%R
Cephalosporin/carbapenem-S^*g*^ (686)								
Cefiderocol	0.06/0.5	≤0.004 to 8	99.7	0.0	99.4	0.6	98.8	0.6
Imipenem-relebactam	0.12/0.25	≤0.03 to 2			100.0	0.0	100.0	0.0
Piperacillin-tazobactam	≤0.06/16	≤0.06 to >128	95.1	2.7			95.1^*b*^	2.7
Ampicillin-sulbactam	2/4	≤0.5 to 64	98.1	0.6			98.1^*b*^	0.6
Ceftazidime	4/8	0.25 to 8	100.0	0.0			100.0^*b*^	0.0
Cefepime	2/8	0.06 to 8	100.0	0.0				
Meropenem	0.25/1	≤0.015 to 2	100.0	0.0	100.0^*c*^ 100.0^*d*^	0.0 0.0	100.0^*b*^	0.0
Colistin	0.5/1	≤0.06 to >8	^*e*^	4.2	95.8	4.2		
Cephalosporin/carbapenem-NS^*h*^ (927)								
Cefiderocol	0.25/2	0.015 to >64	97.3	1.8	94.7	5.3	89.0	5.3
Imipenem-relebactam	>8/>8	≤0.03 to >8			16.4	83.6	16.4	83.1
Piperacillin-tazobactam	>128/>128	≤0.06 to >128	8.9	87.2			8.9^*b*^	87.2
Ampicillin-sulbactam	64/>64	≤0.5 to >64	15.7	75.7			15.7^*b*^	75.7
Ceftazidime	>32/>32	1 to >32	9.3	84.0			9.3^*b*^	84.0
Cefepime	>32/>32	1 to >32	6.7	83.8				
Meropenem	>32/>32	0.06 to >32	15.4	83.9	15.4^*c*^ 15.4^*b*^	84.6 82.6	15.4^*b*^	83.9
Colistin	0.5/>8	0.12 to >8	^*e*^	17.5	82.5	17.5		
MDR (847)^*f*^								
Cefiderocol	0.25/2	0.015 to >64	97.1	2.0	94.2	5.8	88.6	5.8
Imipenem-relebactam	>8/>8	0.06 to >8			8.8	91.2	8.8	90.6
Piperacillin-tazobactam	>128/>128	≤0.06 to >128	2.4	93.9			2.4^*b*^	93.9
Ampicillin-sulbactam	64/>64	1 to >64	8.0	82.8			8.0^*b*^	82.8
Ceftazidime	>32/>32	1 to >32	7.1	89.4			7.1^*b*^	89.4
Cefepime	>32/>32	4 to >32	3.3	88.8				
Meropenem	>32/>32	0.12 to >32	7.8	91.5	7.8^*c*^ 7.8^*d*^	92.2 90.1	7.8^*b*^	91.5
Colistin	0.5/>8	0.12 to >8	^*e*^	19.0	81.0	19.0		
Carbapenemase positive (778)								
Cefiderocol	0.25/2	0.015 to >64	97.4	1.7	94.6	5.4	89.2	5.4
Imipenem-relebactam	>8/>8	0.12 to >8			3.5	96.5	3.5	96.1
Piperacillin-tazobactam	>128/>128	≤0.06 to >128	2.4	96.8			2.4^*b*^	96.8
Ampicillin-sulbactam	64/>64	2 to >64	5.4	86.9			5.4^*b*^	86.9
Ceftazidime	>32/>32	4 to >32	7.3	89.5			7.3^*b*^	89.5
Cefepime	>32/>32	2 to >32	2.2	91.8				
Meropenem	>32/>32	0.25 to >32	3.3	96.5	3.3^*c*^ 3.3^*d*^	96.7 95.9	3.3^*b*^	96.5
Colistin	0.5/>8	0.12 to >8	^*e*^	19.7	80.3	19.7		
Non-carbapenemase (110)								
Cefiderocol	0.25/2	0.015 to >64	95.5	3.6	93.6	6.4	84.5	6.4
Imipenem-relebactam	0.25/8	0.06 to >8			80.9	19.1	80.9	17.3
Piperacillin-tazobactam	64/>128	≤0.06 to >128	28.4	44.0			28.4^*b*^	44.0
Ampicillin-sulbactam	8/64	1 to >64	62.7	22.7			62.7^*b*^	22.7
Ceftazidime	>32/>32	4 to >32	17.3	65.5			17.3^*b*^	65.5
Cefepime	16/>32	2 to >32	18.2	50.0				
Meropenem	1/16	0.12 to >32	74.5	21.8	74.5^*c*^ 74.5^*d*^	25.5 15.5	74.5^*b*^	21.8
Colistin	0.5/2	0.25 to >8	^*e*^	8.2	91.8	8.2		

^*a*^ Criteria as published by CLSI (2023), EUCAST (2023), and US FDA (2023).

^*b*^ Same breakpoints as those published by CLSI.

^*c*^ Results can be considered as susceptible only when treating infections where an increased concentration can be achieved by increased dosing regimens or increased concentration in the site of infections.

^*d*^ Using EUCAST breakpoints for infections other than meningitis.

^*e*^ These breakpoints have been applied to all *Acinetobacter spp*., but are only approved for *Acinetobacter baumannii-calcoaceticus* species complex isolates.

^*f*^ MDR, multi-drug resistant, see Materials and Methods for definitions.

^*g*^ Ceftazidime and cefepime MIC <16 mg/L and imipenem and meropenem MIC <4 mg/L.

^*h*^ Ceftazidime and/or cefepime MIC ≥16 mg/L and imipenem and/or meropenem MIC ≥4 mg/L.

Twenty-seven (1.7%) *A. baumannii* were nonsusceptible to cefiderocol per CLSI criteria (1.7%), and these isolates originated from seven countries with 11 US isolates predominating among the set (Table S5). Acquired carbapenemases were common among these isolates (20/27), with *bla*_OXA-23_ identified in 18 isolates. Acquired MBL and ESBL genes were less common in cefiderocol nonsusceptible *A. baumannii,* with those genes encoding NDM-1 or a variant of PER-type ESBLs (PER-1 or PER-7), identified in three and five isolates, respectively. Three non-*baumannii Acinetobacter* spp. had elevated cefiderocol MIC values including one *A. pittii* (MIC 8 mg/L) and two *A. nosocomialis* isolates (MIC, 8 to >64 mg/L).

## DISCUSSION

This study found ~3% of all Enterobacterales from the US and Europe during 2020 and 2021 were nonsusceptible to imipenem and/or meropenem, with a higher proportion of isolates found in European and adjacent regions (4.1%) compared to those from the USA (1.5%). These findings agree with previous data describing the relatively rare occurrence of these isolates (1.3%) in US hospitals in the period 2016–2020 (14), although a more recent analysis demonstrated an increase in the proportion of carbapenem-resistant Enterobacterales isolates causing infections in US hospitals in 2019–2021. This increase was mostly driven by a rise in the number of *bla*_NDM_-carrying isolates, and nine US isolates included in this study (2020, *n* = 2; 2021, *n* = 7) bore *bla*_NDM_ (15). The proportion of carbapenem-nonsusceptible isolates from Europe found in this study is similar to that previously reported for CRE (3.9%) (16).

The results presented here demonstrate the inhibition by cefiderocol of 96.6% of Enterobacterales (FDA|CLSI breakpoint) possessing different carbapenemase genes. In contrast to the β-lactam/β-lactamase inhibitor combinations tested here, cefiderocol maintained activity against isolates with different combinations of carbapenemase genes, including MBLs. Thus, with the potential increase in the prevalence of MBL-carrying Enterobacterales in the USA, noted here and by others (17), and the already high reported distribution of MBL among CRE in Europe (30%) (16), therapeutic options for treating future infections caused by CRE may be limited to cefiderocol or ceftazidime-avibactam plus aztreonam (3). Replacement of *bla*_KPC_- with *bla*_NDM_-carrying isolates among CRE was reported previously (18–21), and there are few reports of the use of cefiderocol monotherapy or in combination for treating infections caused by MBL-carrying isolates. A recently published *ad hoc* analysis of cefiderocol for treating infections caused by MBL-carrying strains demonstrated favorable results associated with clinical cure, microbiological eradication, and 28-day mortality when compared to the best available therapy and high-dose meropenem (22). This report was followed by similar results of Falcone and Tiseo (23) in a tertiary care hospital in Italy. The favorable treatment data reported with cefiderocol against MBL-producing CRE and its *in vitro* activity shown in this study suggest that cefiderocol may be a good option for the empirical and guided treatment of infections caused by CRE, especially in areas with high prevalence of MBL-producing isolates. Although several recent studies have reported the development of reduced susceptibility to cefiderocol in Enterobacterales isolates harboring MBL-encoding genes, particularly *bla*_NDM_ (24, 25), isolates with these genes represented only 20% of the Enterobacterales isolates displaying cefiderocol nonsusceptible MIC values identified in this study. These results suggest the activity of intrinsic mechanisms, including accumulation of alterations affecting expression and/or activity of the intrinsic AmpC genes (6, 26) and dysregulation or inactivation of cefiderocol transporting proteins, largely contributes to cefiderocol nonsusceptibility and underscores the multifactorial nature of this phenotype in Enterobacterales.

This study shows that a proportion of approximately 18% of all carbapenem-susceptible *E. coli*, *K. pneumoniae*, and *P. mirabilis* isolates collected from 2020 to 2021 in US and European medical centers carried ESBL and/or AmpC plasmid-mediated genes. Although cefiderocol MIC_50/90_ results (MIC_50/90_, 0.5/2 mg/L) against these species sets were eightfold higher than the non-ESBL/pAmpC group (MIC_50/90_, 0.06/0.25 mg/L), cefiderocol still inhibited all but three isolates at the FDA|CLSI breakpoint (≤4 mg/L) or 96.1% at the EUCAST breakpoint (≤2 mg/L). In general, infections caused by Enterobacterales isolates associated with ESBL/pAmpC genes are refractory to many antimicrobial agents. Therefore, the use of carbapenems or other carbapenem-sparing agents, such as ceftazidime-avibactam, is recommended, especially in cases other than urinary tract infections (3).

Approximately 33% of *P. aeruginosa* included in this study were molecularly characterized due to nonsusceptibility to ceftazidime/cefepime and/or imipenem/meropenem and 4.9% (76/1,559) carried carbapenemase genes. Isolates carrying carbapenemase genes in the USA remain rare with reported incidences between 0.1% and 1.9% (27, 28), whereas rates ~20% have been reported among isolates from Europe (29). In general, the results reported here corroborate those findings with only 9.2% of all carbapenemase-producing isolates originating in the USA (1.0% of all molecularly characterized US isolates). Furthermore, the overall absence of carbapenemase-encoding genes among the *P. aeruginosa* isolates characterized in this study underscores the importance of intrinsically derived mechanisms, such as the derepression of the chromosomal *ampC* and intrusion/extrusion control *via* porin mutations and efflux pumps, in governing cephalosporin and carbapenem nonsusceptibility in this species (28, 30). Non-acquired β-lactamase-mediated mechanism(s), such as the Ω-loop and R2-loop mutations in the chromosomal PDC described here, must also play a role in cefiderocol nonsusceptibility as only one of the twelve isolates with a cefiderocol MIC value >4 mg/L carried an acquired ESBL or carbapenemase gene (*bla*_NDM-1_) compared to six isolates with PDC alterations (MIC, 8 to >64 mg/L; modal MIC, 16 mg/L). *P. aeruginosa* isolates with similar PDC alterations have been reported previously in cefiderocol-nonsusceptible isolates (31–33). Further investigations into how PDC alterations work in concert with additional intrinsic mechanisms including variant PBP3s, hyper-efflux, and mutated or dysregulated siderophore import systems to manifest in cefiderocol nonsusceptibility are ongoing.

When tested against cefiderocol, cephalosporin- and/or carbapenem-non-susceptible *P. aeruginosa* were over 95% susceptible (96.5%–99.3%; MIC_50/90_, 0.12/0.5 mg/L), whereas β-lactam-β-lactamase inhibitor combinations, such as imipenem-relebactam, ceftazidime-avibactam, and ceftolozane-tazobactam, showed lower susceptibilities (87.8%–88.8%). Cefiderocol also retained greater activity against the subset of MDR (94.7%–98.6% susceptibility; MIC_50/90_, 0.12/1 mg/L) and DTR (90.0%–97.5% susceptibility; MIC_50/90_, 0.12/1 mg/L) isolates compared to the β-lactam-β-lactamase inhibitor combinations (MDR, 76.5%–78.4% susceptibility; DTR, 55.5%–57.0% susceptibility). The fact that the cefiderocol MIC_50/90_ values obtained against the MDR and DTR subsets were similar to that of the cephalosporin- and/or carbapenem-susceptible group (MIC_50/90_, 0.06/0.25 mg/L) suggests that resistance mechanisms, including those affecting commonly used β-lactam agents, do not greatly affect cefiderocol activity. Isolates nonsusceptible to cefiderocol generally displayed few common molecular characteristics (e.g., MLST, intrinsic *ampC*_PDC_/*bla*_OXA-50_ variants), underscoring the non-clonal nature of this phenotype. Furthermore, these results suggest other mechanisms, such as those governing iron or membrane homeostasis, may contribute in a more conserved manner across diverse *P. aeruginosa* lineages despite the rarity of cefiderocol nonsusceptibility.

*A. baumannii-calcoaceticus* are commonly responsible for pneumonia in hospitalized patients, especially those undergoing mechanical ventilation in ICU (34). Clinical treatment of infections caused by this species is often complicated by the presence of a MDR phenotype (35), which was observed in approximately 53% of all *Acinetobacter* spp. included in this study. The *bla*_OXA-23_ was the most common carbapenem resistance mechanism among *A. baumannii-calcoaceticus* in this study, and its occurrence is comparable to those previously described (36, 37). Similar to *P. aeruginosa*, cefiderocol remained active against diverse β-lactamase genotypes of *A. baumannii-calcoaceticus*, whereas other drugs used for treating infections caused by these pathogens showed limited activity. These results again suggest a lack of cross-resistance between cefiderocol, carbapenems, and newer combinations (38). Among 27 *A*. *baumannii-calcoaceticus* nonsusceptible to cefiderocol, five isolates carried PER-type ESBL genes and most possessed *bla*_OXA-23_, a common feature identified in this study (~36%). PER-type enzymes have been shown to affect the activity of cefiderocol (39), and additional studies are needed to understand the cefiderocol resistance mechanisms in these isolates.

Overall, these data show potent *in vitro* activity of cefiderocol when tested against genotypically and phenotypically diverse resistance subsets of Enterobacterales, *P. aeruginosa,* and *Acinetobacter* spp. causing infection in US and European hospitals collected in 2020 and 2021. Two years after cefiderocol was introduced to the market, its nonsusceptibility remains low and analysis of β-lactamase content showed that it cannot be strictly attributed to any particular β-lactamases other than *bla*_PER_. This is in line with previous studies that demonstrated cefiderocol nonsusceptibility to be a multifactorial phenomenon (6, 40–42). More than 85% of isolates from each subset tested were susceptible to cefiderocol under the current breakpoints, much higher than comparator β-lactam/β-lactamase inhibitor combinations, and this agent also demonstrated uniform activity, which was not observed for other agents used to treat infections caused by these species and their respective resistance subsets. Numerous case reports describing the clinical efficacy of cefiderocol for the treatment of various serious infections were published during the pre- and post-approval period (43–49). The accrual of clinical experiences of cefiderocol for treating serious infections caused by these pathogens combined with the post-marketing *in vitro* surveillance activity data support cefiderocol as an option for treating infections caused by the species groups described in this report. Further clinical experiences and continuous monitoring of activity should establish the clinical relevance of cefiderocol for treating serious infections caused by resistant Gram-negative pathogens.

## MATERIALS AND METHODS

### Clinical isolates

This study comprised a collection of 16,065 Enterobacterales, 4,681 *P*. *aeruginosa,* and 1,613 *Acinetobacter* spp. (1,443 *A*. *baumannii-calcoaceticus* species complex and 170 isolates from 18 other species) collected from various clinical specimens from patients hospitalized in 63 medical centers in all 9 US Census Divisions and 38 sites in Europe, Israel, and Turkey during 2020–2021. This surveillance program followed the design of the SENTRY Antimicrobial Surveillance Program, which monitors the distribution of pathogens causing various community- and hospital-associated infections and antimicrobial resistance. Participating sites followed specific instructions for selecting consecutive and unique isolates (one per patient infection episode) deemed clinically relevant based on local criteria until they reached a target number of pathogens per infection site (50). Sites initially performed bacterial identifications which were confirmed by the monitoring laboratory (JMI Laboratories/Element-Iowa City, Iowa, USA) using standard biochemical methods (e.g., indole production) and/or matrix-assisted laser desorption ionization-time of flight mass spectrometry (Bruker Daltonics, Bremen, Germany), when necessary.

### Antimicrobial susceptibility testing

Susceptibility testing was performed using the Clinical and Laboratory Standards Institute (CLSI) broth microdilution method (51). Frozen-form broth microdilution panels were manufactured by JMI Laboratories (North Liberty, IA, USA) and contained cation-adjusted Mueller-Hinton broth. Susceptibility testing cefiderocol used broth microdilution panels containing iron-depleted media per CLSI guidelines. Panels were quality assured before and during use according to CLSI guidelines (51). MIC values obtained for cefiderocol and comparator agents were interpreted according to CLSI, EUCAST, and the FDA breakpoint criteria, as available (11–13). Isolates displaying nonsusceptible cefiderocol MIC values (≥8 mg/L) were subjected to repeat MIC testing to confirm the initial MIC value. The multi-drug resistance (MDR) phenotype is defined here as those isolates nonsusceptible to at least one agent from ≥3 drug classes as follows: *A. baumannii,* extended-spectrum cephalosporins (cefepime/ceftazidime/ceftriaxone), carbapenems (imipenem/meropenem), piperacillin-tazobactam, fluoroquinolones (ciprofloxacin/levofloxacin), aminoglycosides (amikacin/gentamicin/tobramycin), tetracycline, and ampicillin-sulbactam; *P. aeruginosa,* extended-spectrum cephalosporins (cefiderocol/cefepime/ceftazidime), carbapenems (as above), piperacillin-tazobactam, fluoroquinolones (as above), aminoglycosides (amikacin/tobramycin), and new β-lactam-β-lactamase inhibitor combinations (ceftolozane-tazobactam/ceftazidime-avibactam/imipenem-relebactam); Enterobacterales, extended-spectrum cephalosporins (cefepime/ceftazidime/ceftriaxone), carbapenems (imipenem/meropenem), piperacillin-tazobactam, fluoroquinolones (ciprofloxacin/levofloxacin), and aminoglycosides (amikacin/gentamicin/tobramycin). The difficult-to-treat resistance phenotype in *P. aeruginosa* is defined here as isolates nonsusceptible to ceftazidime, cefepime, piperacillin-tazobactam, imipenem, meropenem, levofloxacin, ciprofloxacin, and aztreonam. MDR and DTR were determined based on CLSI interpretive criteria.

### Molecular characterization

Enterobacterales displaying MIC values ≥2 mg/L for ceftazidime, ceftriaxone, aztreonam, imipenem (excluded for *Proteus mirabilis, P. penneri*, and indole-positive Proteeae) or meropenem were subjected to genome sequencing and screening of acquired β-lactamase genes, as previously described (52). In addition, *P. aeruginosa* and *A. baumannii* with imipenem and/or meropenem MIC ≥4 mg/L or ceftazidime and/or cefepime MIC ≥16 mg/L were also screened for acquired β-lactamase genes.

## Data Availability

Data available upon request.

## SUPPLEMENTAL MATERIAL

Supplemental tables spectrum.01474-24-s0001.pdf Tables S1 to S5.

ASM does not own the copyrights to Supplemental Material that may be linked to, or accessed through, an article. The authors have granted ASM a non-exclusive, world-wide license to publish the Supplemental Material files. Please contact the corresponding author directly for reuse.
