# Supplementary material for: In vitro activity assessment of cefiderocol against Enterobacterales, Pseudomonas aeruginosa, and Acinetobacter spp., including β-lactam nonsusceptible molecularly characterized isolates, collected from 2020 to 2021 in the United States and European hospitals
Source: Microbiol Spectr. 2024 Oct 10;12(11):e01474-24. doi: 10.1128/spectrum.01474-24 (PMC11537082; doi:10.1128/spectrum.01474-24)
Supplement: Supplemental tables — Tables S1 to S5. [file spectrum.01474-24-s0001.pdf]

Table S1. Distribution of species and relevant species groups collected in 2020-2021 from US and European medical centers stratified by infection type.

	**Infection Type**												
	**PIHP**		**BSI**		**UTI**		**IAI**		**SSTI**		**Other**		
**Organism Group/Species**	2020	2021	2020	2021	2020	2021	2020	2021	2020	2021	2020	2021	Total
**Enterobacterales**	**1,920**	**1,921**	**2,561**	**2,644**	**2,637**	**2,541**	**781**	**298**	**76**	**597**	**70**	**19**	**16,065**
Carbapenem Nonsusceptible[a]	77	89	75	81	44	39	16	4	1	11	5	0	442
Carbapenem Susceptible	1,843	1,832	2,486	2,563	2,593	2,502	765	294	75	586	65	19	15,623
E. coli	405	329	1,306	1,336	1,420	1,418	386	142	2	138	1	0	6,883
K. pneumoniae	414	456	447	480	455	384	123	44	6	70	22	1	2,902
P. mirabilis	88	71	134	137	191	175	25	13	4	91	4	0	933
Other Enterobacterales[b]	936	976	599	610	527	525	231	95	63	287	38	18	4,905
P. aeruginosa	**1,153**	**1,271**	**335**	**356**	**222**	**222**	**106**	**105**	**465**	**444**	**1**	**1**	**4,681**
***Acinetobacter* spp.**	**264**	**425**	**127**	**174**	**49**	**100**	**18**	**7**	**135**	**150**	**57**	**107**	**1,613**
A. baumannii-calcoaceticus species complex	253	398	108	148	42	81	13	7	115	126	57	95	1,443
Other *Acinetobacter*[c]	11	27	19	26	7	19	5	0	20	24	0	12	170

Abbr. PIHP, pneumonia in hospitalized patients; BSI, blood stream infection; UTI urinary tract infection; IAI, intra-abdominal infection; SSTI, skin/soft tissue infection

[a] Organisms include *Citrobacter freundii* species complex (7), *C. koseri* (1), *E. cloacae* species complex (41), *Escherichia coli* (12), *Hafnia alvei* (1), *Klebsiella aerogenes* (27), *K. oxytoca* (11), *K. pneumoniae* (305), *Proteus mirabilis* (1), *Providencia rettgeri* (3), *Raoultella* spp. (5), *Serratia liquefaciens* (1), *S. liquefaciens complex* (1), *S. marcescens* (25), *and Yokenella regensburgei* (1).

[b] *Citrobacter amalonaticus / farmeri* (23), *C. braakii* (2), *C. freundii* (18), *C. freundii* species complex (362), *C. koseri* (350), *C. sedlakii* (1), *C. youngae* (1), *Cronobacter sakazakii* (4), *Enterobacter asburiae* (8), *E. cancerogenus* (1), *E. cloacae* (409), *E. cloacae* species complex (718), *E. hormaechei* (7), *E. kobei* (4), *Escherichia hermannii* (1), *E. marmotae* (2), gram-negative rods in the family *Enterobacteriaceae* (2), *Hafnia alvei* (43), *H. alvei/ Hafnia paralvei* (1), *Klebsiella aerogenes* (515), *K. oxytoca* (676), *K. variicola* (145), *Kluyvera ascorbata* (2), *K. cryocrescens* (1), *Leclercia adecarboxylata* (2), *Lelliottia amnigena* (2), *Morganella morganii* (280), *Pantoea agglomerans* (18), *P. ananatis* (2), *P. anthophila* (1), *P. calida* (1), *P. dispersa* (2), *Phytobacter diazotrophicus* (1), *Pluralibacter gergoviae* (8), *Proteus hauseri* (5), *P. penneri* (20), *P. vulgaris* (74), *P. vulgaris* group (53), *Providencia rettgeri* (73), *P. stuartii* (66), *Pseudocitrobacter faecalis* (1), *Rahnella aquatilis* (3), *Raoultella ornithinolytica* (42), *R. planticola* (5), *Serratia fonticola* (1), *S. liquefaciens* (22), *S. liquefaciens* complex (6), *S. marcescens* (872), *S. odorifera* (4), *S. rubidaea* (3), unspeciated *Citrobacter* (5), unspeciated *Klebsiella* (1), unspeciated *Pantoea* (3), unspeciated *Providencia* (4), unspeciated *Raoultella* (28), and *Yersinia enterocolitica* (1).

[c] Includes *Acinetobacter beijerinckii* (1), *A. bereziniae* (19), *A. courvalinii* (6), *A. dispersus* (1), *A. gerneri* (1), *A. guillouiae* (2), *A. gyllenbergii* (2), *A. haemolyticus* (2), *A. johnsonii* (12), *A. junii* (20), *A. lwoffii* (10), *A. proteolyticus* (6), *A. radioresistens* (26), *A. schindleri* (3), *A. soli* (3), *A. ursingii* (40), *A. variabilis* (2), *A. vivianii* (3), and unspeciated *Acinetobacter* (11).

Table S2. Distribution of species and relevant species groups collected in 2020-2021 from US and European medical centers stratified by geographic origin.

	**Enterobacterales**				***Pseudomonas***		***Acinetobacter* spp.**				
	Carbapenem Susceptible		Carbapenem Nonsusceptible		*P. aeruginosa*		*A. baumannii-calcoaceticus* complex		Other spp.		
**Continent-Country**	2020	2021	2020	2021	2020	2021	2020	2021	2020	2021	Total
**Europe**	**3,825**	**3,597**	**169**	**148**	**1,213**	**1,227**	**340**	**515**	**26**	**50**	**11,110**
Belgium	106	130	2	3	29	22	2	3	1	1	299
Czech Republic	83	79	0	1	32	10	2	1	1	0	209
France	325	413	4	2	90	160	1	6	2	3	1,006
Germany	739	539	14	6	180	142	70	32	4	9	1,735
Greece	130	98	21	31	47	45	48	50	0	0	470
Hungary	96	68			30	36	3	12	0	0	245
Ireland	169	127	1	0	24	24	2	3	2	2	354
Israel	102	188	5	3	38	94	19	133	0	5	587
Italy	496	511	39	22	196	218	47	117	9	17	1,672
Poland	55	82	23	30	75	36	39	32	0	0	372
Portugal	102	140	3	3	27	42	12	9	0	2	340
Romania	55	31	2	5	17	10	3	4	0	0	127
Slovenia	151	175	1	0	40	35	0	6	1	0	409
Spain	369	379	21	6	190	173	6	8	0	5	1,157
Sweden	193	133			37	22	1	1	1	0	388
Switzerland	163	106	1	0	34	34	5	28	1	3	375
Turkey	209	174	27	34	68	87	78	65	2	1	745
UK	282	224	5	2	59	37	2	5	2	2	620
**North America (USA)**	**4,002**	**4,199**	**49**	**76**	**1,069**	**1,172**	**248**	**340**	**36**	**58**	**11,249**
**Total**	**7,827**	**7,796**	**218**	**224**	**2,282**	**2,399**	**588**	**855**	**62**	**108**	**22,359**

Table S3. Molecular characterization of Enterobacterales isolates with cefiderocol MIC values >4 mg/L collected from 2020-2021 from US and European medical centers.

Organism	MLST	Year	Country	FDC	IMI	MEM	β-lactamase Content
K. pneumoniae	323	2020	Italy	>64	0.5	0.12	KPC-31, CTX-M-15, SHV-1
K. aerogenes	176	2021	USA	>64	4	2	AmpC (G123E,Q140K,S189G,P299S,E301G,V303L,N322K,S327P,V328L,N366Y)
K. aerogenes	9-like	2020	Italy	32	2	1	KPC-3, AmpC
E. hormaechei	66-like	2020	USA	32	0.5	0.06	ACT-16, SHV-12
K. pneumoniae	37	2021	Israel	32	0.5	4	CTX-M-15, SHV-11 (C7G)
E. roggenkampii	165	2021	Poland	16	1	0.25	MIR-11, CTX-M-15
E. kobei	125	2021	USA	16	0.25	0.12	ACT-28 (V318E)
E. hormaechei	108	2020	France	8	0.5	0.12	ACT-108
K. aerogenes	16	2020	Germany	8	2	0.5	AmpC
E. hormaechei	544	2020	Poland	8	1	2	ACT-25, CTX-M-15, OXA-1
K. pneumoniae	11	2020	Poland	8	>8	>32	NDM-1, CTX-M-15, OXA-1, SHV-11
K. pneumoniae	14	2020	Turkey	8	>8	>32	NDM-1, OXA-48, CTX-M-15, OXA-1, SHV-11
C. amalonaticus	NT	2020	USA	8	0.25	0.12	FOX-5
E. cloacae	461	2020	USA	8	0.25	0.06	ACT-17, SHV-5
E. cloacae	461	2020	USA	8	0.25	0.06	ACT-17, SHV-12
E. coli	101	2020	USA	8	0.119	0.06	CTX-M-55
E. hormaechei	527	2020	USA	8	0.25	0.03	ACT-60, SHV-12
K. pneumoniae	2004	2020	USA	8	4	16	CTX-M-15, OXA-1, SHV-168
S. marcescens	NT	2020	USA	8	0.5	0.06	SRT (Q21H, E73Q, N102K, N142D, D265E, I299V, V372I, R378H)
K. pneumoniae	258	2021	Greece	8	>8	>32	KPC-2, VEB-1, OXA-10, SHV-11
E. coli	167	2021	Italy	8	>8	>32	NDM-5, CTX-M-32
E. hormaechei	108	2021	Poland	8	4	4	ACT-17(T21A,V145I,P189S,S267T,E305G,T362K), CTX-M-15, OXA-1
K. pneumoniae	11	2021	Poland	8	>8	>32	NDM-1, CTX-M-15, OXA-1, SHV-11
K. pneumoniae	152	2021	Poland	8	1	2	CTX-M-15, SHV-1
E. coli	10	2021	Turkey	8	0.119	0.06	CTX-M-15, OXA-1
K. pneumoniae	14	2021	Turkey	8	>8	>32	NDM-1, OXA-48, CTX-M-15, OXA-1, SHV-28
K. pneumoniae	414	2021	Turkey	8	8	16	NDM-1, CMY-6, CTX-M-15
E. hormaechei	132-like	2021	USA	8	1	1	ACT-16
E. hormaechei	144	2021	USA	8	0.5	0.03	ACT-25 (L313_L313del)
E. kobei	191	2021	USA	8	0.25	0.06	ACT-52, SHV-12

Abbreviations: MLST, multi-locus sequence type; FDC, cefiderocol; IMI, imipenem; MEM, meropenem; NT, non-typeable

Table S4. Molecular characterization of *P. aeruginosa* with cefiderocol MIC values >4 mg/L collected from 2020-2021 from US and European medical centers.

MLST	Year	Country	FDC	IMI	MEM	β-lactamase Content
875-like	2021	France	>64	4	16	OXA-50, PDC-97(T96I,E247K)
2000-like	2020	Italy	32	4	4	OXA-488, PDC-337
274-like	2020	France	16	1	0.5	OXA-486, PDC-230 (G183D,E247K,N273T)
274-like	2020	France	16	>8	16	OXA-486, PDC-540 (A239T)
447	2020	USA	16	2	32	OXA-395, PDC-452 (D245N,L320S)
1248	2020	USA	16	4	16	OXA-395, PDC-336
730-like	2020	Germany	8	>8	32	OXA-50, PDC-8
235	2021	Spain	8	1	2	OXA-488, PDC-35
773	2021	Turkey	8	>8	>32	NDM-1, OXA-395, PDC-16
447	2020	USA	8	2	4	OXA-395, PDC-452 (D245N,L320S)
1985-like	2020	USA	8	8	4	OXA-1028, PDC-368 (P180L)
3694	2020	USA	8	8	8	OXA-904, PDC-97 (P243L)

Abbreviations: MLST, multi-locus sequence type; FDC, cefiderocol; IMI, imipenem; MEM, meropenem

Table S5. Molecular characterization of *A. baumannii-calcoaceticus* species complex isolates with cefiderocol MIC values >4 mg/L collected from 2020-2021 from US and European medical centers.

MLST	Year	Country	FDC	IMI	MER	β-lactamase Content
2	2020	Turkey	>64	>8	>32	PER-1, OXA-23, OXA-66, ADC-25
2	2020	Turkey	>64	>8	>32	PER-1, OXA-23, OXA-66, ADC-25
2	2020	Turkey	>64	>8	>32	PER-7, OXA-23, OXA-66, ADC-30
2	2020	Turkey	>64	>8	>32	PER-7, OXA-23, OXA-66, ADC-30
2	2021	USA	>64	8	16	OXA-113, SHV-12, ADC-30
108	2021	USA	>64	>8	>32	OXA-23, OXA-80, ADC-33
71[a]	2021	USA	>64	0.25	0.12	ADC-130
2	2021	Italy	32	>8	>32	OXA-23, OXA-82, ADC-33
2	2021	USA	32	8	16	OXA-82, ADC-33
2	2020	Italy	16	>8	32	OXA-23, OXA-82, ADC-33
2	2020	Italy	16	>8	>32	OXA-23, OXA-82, ADC-33 (G343V)
2	2020	USA	16	>8	32	OXA-23, OXA-82, ADC-33 (Q315_M318del)
2	2020	USA	16	>8	>32	OXA-23, OXA-66, ADC-177 (G247S)
499	2020	USA	16	>8	>32	OXA-24, OXA-95, ADC-222
2	2021	Italy	16	>8	>32	OXA-23, OXA-82, ADC-33
2	2021	Poland	16	1	1	OXA-66, ADC-30
2	2021	Turkey	16	>8	>32	PER-1, OXA-23, OXA-66, ADC-25
2	2020	Greece	8	>8	>32	OXA-23, OXA-66, ADC-177 (G247S)
1	2020	Italy	8	>8	>32	OXA-23, OXA-69, ADC-33 (N342S)
214[b]	2020	Switzerland	8	0.25	0.5	OXA-500
2	2020	USA	8	>8	>32	OXA-23, OXA-82, ADC-33
2	2020	USA	8	>8	16	OXA-82, ADC-33
395[a]	2020	USA	8	0.25	0.5	ADC-239
2	2021	Israel	8	>8	>32	NDM-1, OXA-23, OXA-66, ADC-73
85	2021	Israel	8	>8	>32	NDM-1, OXA-94, ADC-80
2	2021	Italy	8	>8	>32	OXA-23, OXA-66, ADC-56
2	2021	USA	8	>8	>32	NDM-1, OXA-23, OXA-66, ADC-30

Abbreviations: MLST, multi-locus sequence type; FDC, cefiderocol; IMI, imipenem; MEM, meropenem

[a] *A. nosocomialis.*

[b] *A. pittii.*
